# The NeuroD6 Subtype of VTA Neurons Contributes to Psychostimulant Sensitization and Behavioral Reinforcement

**DOI:** 10.1523/ENEURO.0066-19.2019

**Published:** 2019-06-07

**Authors:** Zisis Bimpisidis, Niclas König, Stefanos Stagkourakis, Vivien Zell, Bianca Vlcek, Sylvie Dumas, Bruno Giros, Christian Broberger, Thomas S. Hnasko, Åsa Wallén-Mackenzie

**Affiliations:** 1 Department of Organismal Biology, Uppsala University, 75236 Uppsala, Sweden; 2 Department of Neuroscience, Karolinska Institutet, 17177 Stockholm, Sweden; 3 Department of Neurosciences, University of California, San Diego, La Jolla, CA; 4 Research Service VA San Diego Healthcare System, San Diego, CA 92161; 5 Oramacell, 8 Rue Grégoire de Tours, 75006 Paris, France; 6 Institut National de la Santé et de la Recherche Médicale, INSERM UMRS 1130; Centre National de la Recherche Scientifique, Unité Mixte de Recherche 8246; Sorbonne University Université Pierre-et-Marie-Curie, Neurosciences Paris-Seine, F-75005, Paris, France; 7 Douglas Mental Health University Institute 6875 LaSalle blvd, Verdun (Qc), H4H 1R3, Montreal, Canada; 8 Department of Psychiatry, McGill University, Montreal, Canada

**Keywords:** accumbens, dopamine, mouse genetics, optogenetics, reward, ventral tegmental area

## Abstract

Reward-related behavior is complex and its dysfunction correlated with neuropsychiatric illness. Dopamine (DA) neurons of the ventral tegmental area (VTA) have long been associated with different aspects of reward function, but it remains to be disentangled how distinct VTA DA neurons contribute to the full range of behaviors ascribed to the VTA. Here, a recently identified subtype of VTA neurons molecularly defined by NeuroD6 (NEX1M) was addressed. Among all VTA DA neurons, less than 15% were identified as positive for NeuroD6. In addition to dopaminergic markers, sparse NeuroD6 neurons expressed the vesicular glutamate transporter 2 (*Vglut2*) gene. To achieve manipulation of NeuroD6 VTA neurons, NeuroD6(NEX)-Cre-driven mouse genetics and optogenetics were implemented. First, expression of vesicular monoamine transporter 2 (VMAT2) was ablated to disrupt dopaminergic function in NeuroD6 VTA neurons. Comparing *Vmat2^lox/lox;NEX-Cre^* conditional knock-out (cKO) mice with littermate controls, it was evident that baseline locomotion, preference for sugar and ethanol, and place preference upon amphetamine-induced and cocaine-induced conditioning were similar between genotypes. However, locomotion upon repeated psychostimulant administration was significantly elevated above control levels in cKO mice. Second, optogenetic activation of NEX-Cre VTA neurons was shown to induce DA release and glutamatergic postsynaptic currents within the nucleus accumbens. Third, optogenetic stimulation of NEX-Cre VTA neurons *in vivo* induced significant place preference behavior, while stimulation of VTA neurons defined by Calretinin failed to cause a similar response. The results show that NeuroD6 VTA neurons exert distinct regulation over specific aspects of reward-related behavior, findings that contribute to the current understanding of VTA neurocircuitry.

## Significance Statement

Reward-related behavior is complex and its dysfunction is implicated in many neuropsychiatric disorders, including drug addiction. Midbrain dopamine (mDA) neurons of the ventral tegmental area (VTA) are crucial for reward behavior, but due to recently uncovered heterogeneity, it remains to be fully resolved how they regulate reward responsiveness and how their dysfunction might contribute to disease. Here we show that the recently described NeuroD6 (NEX) subtype of VTA DA neurons is involved in psychostimulant sensitization and that optogenetic stimulation of NEX-Cre VTA neurons induces DA release, glutamatergic postsynaptic currents, and real-time place preference behavior. NeuroD6 VTA neurons thus exert distinct regulation over specific aspects of reward-related behavior, findings that contribute to the current understanding of VTA neurocircuitry.

## Introduction

The midbrain dopamine (mDA) system mediates a diverse spectrum of behaviors and their dysfunction is correlated with a range of severe behavioral disorders including substance use disorder, schizophrenia, ADHD and Parkinson’s disease (PD). Consequently, therapies based on modulating the activity of the mDA system are commonly prescribed, however, due to their unselective nature, current treatments often fail to alleviate symptoms and instead cause adverse effects (Weintraub, 2008; Divac et al., 2014). One reason for the lack of successful treatment is incomplete understanding of the underlying neurobiology. Indeed, it is increasingly understood that the mDA system is highly heterogeneous (for review, see Pupe and Wallén-Mackenzie, 2015; Morales and Margolis, 2017). Beyond the classical separation into the ventral tegmental area (VTA) and substantia nigra pars compacta (SNc), with VTA projections to cortical and limbic target areas and SNc projections to the dorsal striatum subserving cognitive/affective and motor functions, respectively (Björklund and Dunnett, 2007), a higher level of complexity is now being unfolded: afferent and efferent projections, electrophysiological patterns, capacity for glutamate or GABA co-release, and responsiveness to appetitive or aversive stimuli are some of the properties that distinguish mDA neurons from each other (Lammel et al., 2011; Beier et al., 2015; Menegas et al., 2015; Faget et al., 2016).

Likely coupled to this functional diversity is a complex diversity in molecular identity. Microarray-based analyses have identified gene expression patterns enriched in VTA over SNc DA neurons (Chung et al., 2005; Greene et al., 2005; Viereckel et al., 2016) while single cell profiling has begun to identify combinatorial gene expression patterns that molecularly define subtypes of mDA neurons (Poulin et al., 2014; La Manno et al., 2016; Hook et al., 2018). Based on this new knowledge, intersectional genetic approaches were recently described in which the distinct projection pathways of several newly defined subtypes of mDA neurons were identified (Poulin et al., 2018). By forwarding the current knowledge toward molecularly defined, and thus targetable, subtypes of mDA neurons with distinct projection patterns, these recent advances enhance the possibility of improving selectivity in treatment of dopaminergic disorders. However, a key issue that remains to be resolved is how each molecularly defined subtype of DA neuron contributes to the complex range of behaviors ascribed to the mDA system.

The gene encoding the transcription factor NeuroD6 (also known as NEX1M) has recently gained attention due to its selective expression within subsets of VTA DA neurons while being excluded from the SNc (Viereckel et al., 2016; Khan et al., 2017; Kramer et al., 2018). VTA DA neurons are of particular interest for several reasons. First, the importance of VTA DA neurons in several aspects of behavioral reinforcement and conditioning has been established through classical studies (for review, see Di Chiara and Bassareo, 2007; Ikemoto, 2007), and more recently, by the use of optogenetics (Tsai et al., 2009; Kim et al., 2012; Ilango et al., 2014; Pascoli et al., 2015). However, detailed knowledge of the exact nature of those particular DA neurons that contribute to each of these complex behaviors remains elusive. Second, medial DA neurons mediate the most potent responsiveness to addictive drugs via their projection to the nucleus accumbens shell (NAcSh; Ikemoto and Bonci, 2014). The possibility to ascribe specific aspects of drug responses to a distinct subtype of VTA DA neurons would therefore enhance the understanding of addictive behavior. Third, certain VTA neurons show resistance to degeneration in PD (Brichta and Greengard, 2014); however, depending on their role in behavioral regulation, surviving VTA neurons might contribute to non-motor symptoms including behavioral addictions (Cenci et al., 2015).

While *NeuroD6*-expressing DA neurons were recently identified as neuroprotected in experimental PD (Kramer et al., 2018), the potential role of this newly described subtype of VTA neurons in behavioral regulation has remained unexplored. Here, we implemented NeuroD6-Cre mice (also known as NEX-Cre) to create opportunities for targeting and manipulation of the NeuroD6 subtype VTA neurons. We show that gene targeting of vesicular monoamine transporter 2 (VMAT2) within this particular DA neuron subtype elevated the locomotor response to psychostimulants while activation of NeuroD6-Cre neurons by optogenetic stimulation in the medial VTA induced DA release and glutamatergic postsynaptic responses in the NAcSh. *In vivo* optogenetic activation of the NeuroD6-Cre VTA subpopulation in a real-time place preference (RT-PP) failed to trigger a conditioned response (CR) but induced place preference upon direct stimulation. These results advance the current understanding of the VTA circuitry by identifying discrete aspects of reward-related behavior correlated with the NeuroD6 subtype VTA neurons.

## Materials and Methods

### Mice

Mice were provided with food and water ad libitum and housed according to Swedish legislation (Animal Welfare Act SFS 1998:56) and European Union legislation (Convention ETS 123 and Directive 2010/63/EU). Mice of either sex were used. Experiments were conducted with permission from the local Animal Ethical Committees. DAT-Cre (Ekstrand et al., 2007), Vglut2-Cre (Borgius et al., 2010), Calb2-Cre (The Jackson Laboratory, RRID:MGI_4365741), and NeuroD6-Cre/NEX-Cre (Goebbels et al., 2006) transgenic mice were bred with C57BL/6N Tac wild-type mice (Taconic) for optogenetics-based experiments. NEX-Cre mice were also bred with *Vmat2^lox/lox^* mice, in which exon 2 of the *Vmat2* gene is flanked by LoxP sites (Narboux-Nême et al., 2011) to generate conditional knock-out (cKO; *Vmat2^lox/lox;NEX-Cre-tg^*) mice in which *Vmat2* exon 2 is ablated on NEX-Cre-mediated recombination of LoxP sites. Littermate mice negative for the NEX-Cre-transgene served as control mice (*Vmat2^lox/lox;NEX-Cre-wt^*: Ctrl; illustrated in Fig. 2*A*). Mice were genotyped by PCR using the following primer sequences: Cre (applies to DAT-Cre, NEX-Cre, and Calb2-Cre): 5’-ACG AGT GAT GAG GTT CGC AAG A-3’; 5’-ACC GAC GAT GAA GCA TGT TTA G-3’; Vglut2-Cre: 5’-TTG CAT CGC ATT GTC TGA GTA G-3’; 5’-TTC CCA CAC AAG ATA CAG ACT CC-3’; Vmat2Lox: 5’-GAC TCA GGG CAG CAC AAA TCT CC-3’; 5’-GAA ACA TGA AGG ACA ACT GGG ACC C-3’.

### *In situ* hybridization (ISH)

For ISH using radioactive oligoprobes, the following probes sequences were used:

NeuroD6: NM_009717.2; bases 99–132, 933–966, and 1256–1288

Th: NM_009377.1; bases 774–807, 272–305, and 1621–1655

Vmat2exon1: NM_172523.3; bases 18–51 and 83–116

Vmat2exon2: NM_172523.3; bases 201–237 and 240–276

Oligoprobes were 3’ end-labeled with [alpha-^35^S]dATP using terminal deoxynucleotidyl transferase at a specific activity of 5 × 10^8^ d.p.m./µg. Sections were fixed in 3.7% formaldehyde in PBS for 1 h, washed in PBS, rinsed in water, dehydrated in 70% ethanol and air dried. Hybridization was conducted at 42°C for 16 h in hybridization medium (Oramacell) containing the labeled antisense oligonucleotides (3.10^5^ cpm/100 µl). Sections were washed to a final stringency of 0.5 SSC at 53°C, dehydrated in ethanol, air-dried and exposed to Fujifilm BioImaging Analyzer BAS-5000 for 15 d.

For double and triple ISH using riboprobes [fluorescent ISH (FISH) or combined FISH/brightfield ISH (FISH/ISH)], the following probes sequences were used:

Calb2: NM_007586.1; bases 80–793

Dat (Slc6a3): NM_012694.2; bases 1015–1938

NeuroD6: NM_009717.2; bases 635–1419

Th: NM_009377.1; bases 456–1453

Vglut2 (Slc17a6): NM_080853.3; bases 2315–3244

Viaat (Slc32a1): NM_009508.2; bases 649–1488

Vmat 2 probe 1: Vmat2: NM_0130331.1 (rat); bases 701–1439 (corresponds to exon 6–15 of mouse sequence NM_172523.3)

Vmat2 probe 2: NM_172523.3; bases142–274, i.e., the whole exon 2.

Detection of Th, Dat, Vglut2, Viaat, Calb2, NeuroD6 mRNA, and Vmat2 probe 1 and probe 2 mRNA in brain tissue using ISH was performed following a previously published protocol (Viereckel et al., 2016). Briefly, mice were sacrificed and brains dissected. Coronal cryosections were prepared, air-dried, fixed in 4% paraformaldehyde and acetylated in 0.25% acetic anhydride/100 mM triethanolamine (pH 8) followed by hybridization for 18 h at 65°C in 100 μl of formamide-buffer containing 1 μg/ml digoxigenin (DIG)-labeled probe for colorimetric detection or 1 μg/ml DIG- or 1 μg/ml fluorescein-labeled probes for fluorescent detection. Sections were washed at 65°C with SSC buffers of decreasing strength, and blocked with 20% FBS and 1% blocking solution. For colorimetric detection, DIG epitopes were detected with alkaline phosphatase-coupled anti-DIG fab fragments at 1:500 and signal developed with NBT/BCIP. For fluorescent detection, sections were incubated with HRP-conjugated anti-fluorescein antibody at 1:1000 concentration (Roche catalog #11426346910, RRID:AB_840257). Signals were revealed with the TSA kit (PerkinElmer catalog #NEL749A001KT) using biotin tyramide at 1:75 concentration followed by incubation with neutravidin Oregon Green conjugate at 1:750 (Invitrogen catalog #A-6374, RRID:AB_2315961). HRP-activity was stopped by incubation of sections in 0.1 M glycine and 3% H_2_O_2_. DIG epitopes were detected with HRP-conjugated anti-DIG antibody at 1:1000 (Roche catalog #11207733910, RRID:AB_514500) and revealed with TSA kit (PerkinElmer catalog #NEL744A001KT) using Cy3 tyramide at 1:200. For triple FISH, TH mRNA was detected with dinitrophenyl (DNP)-labeled probe; NeuroD6 mRNA with DIG-labeled probe and Vglut2 mRNA with fluorescein-labeled probe. The protocol was the same as described above until revelation: DIG epitopes were detected with HRP anti-DIG fab fragments at 1:3000 and revealed using Cy3 tyramide at 1:50 followed by glycine and H_2_O_2_ treatment. Fluorescein epitopes were detected with HRP anti-fluorescein fab fragments at 1:5000 and revealed using Cy2 tyramide at 1:250 by glycine and H_2_O_2_ treatment. DNP epitopes were detected with HRP anti-DNP fab fragments at 1:1000 and revealed using Cy5 tyramide at 1:50, followed by incubation with DAPI. Fluorophore tyramides were synthetized as previously described (Hopman et al., 1998). All slides were scanned and analyzed on NanoZoomer 2.0-HT Ndp2.view (Hamamatsu). Stereotaxic reference atlases (Franklin and Paxinos, 2008; Fu et al., 2012) were used to outline anatomic borders.

### Validation of NEX-Cre-mediated recombination of floxed *Vmat2* exon 2

Upon genotyping, PCR-validated *Vmat2^lox/lox;NEX-Cre-tg^*(cKO) and *Vmat2^lox/lox;NEX-Cre-wt^* (Ctrl) mice were sacrificed and brains analyzed by ISH to verify NEX-Cre-driven recombination of the floxed exon 2 of the *Vmat2* gene in cKO mice. Littermate Ctrl mice were used to validate wild-type Vmat2 mRNA. A Vmat2 mRNA two-probe approach was implemented to visualize cells positive for wild-type Vmat2 mRNA and cells positive for a truncated Vmat2 mRNA generated on NEX-Cre-driven recombination of the floxed *Vmat2* exon 2. Probe 1 (green) was designed for detection of Vmat2 mRNA derived from exon 6–15 and probe 2 (blue) for detection of mRNA from exon 2. In control mice, both probe 1 and probe 2 can bind their target mRNA (wild-type Vmat2 mRNA). Combination of probe 1 and probe 2 gives rise to combined blue and green labeling in wild-type DA neurons. In cKO mice, *Vmat2* exon 2 will be deleted specifically in cells expressing the NEX-Cre transgene, leading to production of Vmat2 mRNA missing exon 2 but maintaining exons 6–15. In Vmat2-expressing cells that do not express the NEX-Cre transgene in cKO mice, wild-type Vmat2 mRNA will be produced. *Vmat2*-targeted cells can thus be identified based on lack of blue color (probe 2) and presence of green color only (probe 1). Thus, using the Vmat2 mRNA two-probe-approach, the color shift from complete overlap of blue and green color in Ctrl mice to the presence of green-only cells in cKO mice is used to verify Cre-LoxP-mediated cKO of the *Vmat2* gene.

### Immunohistochemistry

Detection of TH and eYFP proteins took place according to standard immunohistochemical protocols using primary antibodies [mouse anti-TH (1:1000, Millipore catalog #MAB318, RRID:AB_2201528), chicken anti-GFP (1:1000, Abcam catalog #ab13970, RRID:AB_300798)]. After overnight incubation, primary antibodies were removed and sections were incubated in specific fluorophore-conjugated secondary antibodies (donkey anti-mouse Cy3, Millipore catalog #AP192C, RRID:AB_11214096, donkey anti-chicken A488, Jackson ImmunoResearch catalog #703-545-155, RRID:AB_2340375, both 1:500). Upon rinses, slides were coverslipped using Fluoromount Aqueous mounting medium (Sigma-Aldrich catalog #F4680). For bright-field detection of TH, the peroxidase-based method (ABC kit; Vector Laboratories catalog #PK-4001, RRID:AB_2336810) with DAB chromogen was used. Quantifications were done manually on three mice per group. A stereotaxic atlas (Franklin and Paxinos, 2008) was used to outline anatomic borders.

### Behavioral analysis

*Vmat2^lox/lox;NEX-Cre-tg^* cKO and *Vmat2^lox/lox;NEX-Cre-wt^* Ctrl mice were analyzed in the following behavioral tests.

#### Baseline locomotion

Spontaneous locomotion and habituation in a novel environment were monitored for 30 min upon placing the mice in Makrolon polycarbonate boxes containing 1.5-cm bedding and a transparent Plexiglas lid. Locomotor behavior of the mice was recorded by the EthovisionXT software (Noldus, RRID:SCR_000441).

#### Sucrose preference test

Preference to sucrose was assessed in the home cage of the mice. The mice were housed individually in cages containing two drinking bottles. After 48 h of habituation to the experimental set up, they were presented to one bottle of tap water and one of sucrose solution (1%, 3%, and 10%) that were replaced and weighted every 24 h. Each concentration was tested twice and the position of the bottles was alternated to avoid side bias.

#### Ethanol preference test

Individually housed mice had access to one bottle of tap water and one of alcohol solution (3%, 6%, and 10%) that were replaced and weighted every 24 h. Each concentration of ethanol was tested four times.

#### Cocaine-induced locomotion

Mice were placed in Makrolon polycarbonate boxes containing 1.5-cm bedding and a transparent Plexiglas lid and their locomotor behavior was recorded 30 min before and 60 min after injection of saline or cocaine (5, 10, and 20 mg/kg, i.p.) on four consecutive days. Locomotor behavior of the mice was recorded by the EthovisionXT software (Noldus, RRID:SCR_000441).

#### Amphetamine sensitization

Upon habituation, mice received a saline injection (day 1) followed by 4 d of amphetamine injections (days 2–5, 3 mg/kg, i.p.) followed by a last injection on day 17. Locomotion was recorded 30 min before and 1.5 h after injection. Locomotor behavior of the mice was recorded by the EthovisionXT software (Noldus, RRID:SCR_000441).

#### Conditioned-placed preference (CPP)

An apparatus (Panlab, Harvard Apparatus) consisting of two-main compartments [20 cm (W) × 18 cm (L) × 25 cm (H)] with distinct wall and floor texture patterns and one connecting, transparent compartment (20 × 7 × 25 cm) was used. The CPP procedure was conducted throughout 6 d. Firstly, during the pre-test, the mice were placed in the apparatus and left to freely explore. This session was used to assess initial preferences and to calculate the preference score (see below). During the next four consecutive conditioning days, the mice were constrained in one of the two main compartments and received drug injections (cocaine, 20 mg/kg or amphetamine, 3 mg/kg; i.p.) in the least preferred compartment or saline injections in the opposite one. The conditioning sessions were repeated twice a day [morning (A.M.), afternoon (P.M.)] and the treatment was alternated between days. Thus, the mice received in total four injections of saline and four injections of the drug, counterbalanced between sessions and genotypes. On the test day, the mice were placed again in the apparatus and were let to freely explore. The preference score was calculated by subtracting the time in seconds the animal spent in the drug-paired compartment during pre-test from the time spent in the same compartment during the test (ΔSec). All sessions lasted 30 min, and the locomotor behavior of the mice was recorded by the EthovisionXT software (Noldus, RRID:SCR_000441).

### Stereotaxic injections

Optogenetic viruses were purchased from University of North Carolina, Vector Core Facilities. DAT-Cre, Vglut2-Cre, Calb2-Cre and NEX-Cre mice (more than eight weeks; >20 g) were deeply anesthetized with isofluorane and received infusions of 300 nl of *AAV5-EF1a-DIO-ChR2(H134)-eYFP* or *AAV5-EF1a-DIO-eYFP-WPREpA* in the right VTA (AP: –3.45 mm, L: –0.2 mm, V: –4.4 mm according to Franklin and Paxinos, 2008) at 100 nl min^−1^ flow rate. For behavioral analysis, an optic fiber was implanted and stabilized above the right VTA (AP: –3.4 mm, ML: –0.3 mm, DV: –4.0 mm) using anchor screws and dental cement. A subset of NEX-Cre mice was injected bilaterally with *AAV5-EF1a-DIO-ChR2(H134)-eYFP* before fiber implantation. After postmortem histological validation, mice with limited transfection in the VTA and/or misplaced optic fiber were excluded from statistical analysis.

### Imaging, cell counting, and analysis of projection target areas

#### Quantification of FISH

Manual counting of cells expressing mRNAs of interest was performed in two to three mice per probe pair with Th mRNA as reference for outline of the VTA and Th, Dat, Viaat, or Vglut2 mRNA as reference for distinct cell soma. A signal for a particular mRNA was considered as specific for a particular cell when five contiguous fluorescent puncta were present within the outline of the cell soma.

#### Quantification of immunohistochemistry

Sections of Calb2-Cre and NEX-Cre mice injected with *AAV5-EF1a-DIO-ChR2(H134)-eYFP* containing the VTA (–3.28 to –3.80 mm from bregma according to Franklin and Paxinos, 2008) were immunostained for eYFP and TH as described above. Z-stacks in four different positions within the VTA, (VTA1–VTA4, of which VTA1 and VTA3 represented medial VTA and VTA2 and VTA4 lateral VTA on two different bregma levels), were acquired using a Zeiss Confocal microscope (LSM 700, 20× magnification). Co-labeling of YFP and TH was identified for each fluorescent channel and counted manually using the ImageJ software (RRID:SCR_003070). A minimum of three mice of each genotype was processed and analyzed.

#### Analysis of projection areas

Fluorescent microscopy (Zeiss Confocal microscope) was used to detect eYFP-positive fibers in sections derived from the whole brain of NEX-Cre, Calb2-Cre, DAT-Cre, and NEX-Cre mice injected into the VTA with *AAV5-EF1a-DIO-ChR2(H134)-eYFP*. A minimum of two mice of each genotype was analyzed by two persons blind to the genotype of the mice.

### Fast-scan cyclic voltammetry (FSCV) in slices

For DA recordings in terminal areas upon photostimulation, DAT-Cre, Calb2-Cre, and NEX-Cre mice were injected with *AAV5-EF1a-DIO-ChR2(H134)-eYFP* or *AAV5-EF1a-DIO-eYFP-WPREpA* as described above.

#### Carbon fiber microelectrodes

Carbon fiber working electrodes were fabricated by aspirating 7-μm diameter carbon fibers (Cytec Engineered Materials) into borosilicate glass capillaries (1.2 mm O.D., 0.69 mm I.D., Sutter Instrument Co). Capillaries were adjusted (Sutter Instrument, P-97) and sealed with epoxy (EpoTek 301, Epoxy Technology). Electrodes were tested on bath applications of known concentrations of DA. Only electrodes showing good reaction kinetics (current vs time plots, and current vs voltage plots) were used.

#### FSCV

A Dagan Chem-Clamp potentiostat (Dagan Corporation) and two data acquisition boards (PCI-6221, National Instruments) run by the TH 1.0 CV program (ESA) were used to collect all electrochemical data. Cyclic voltammograms were obtained by applying a triangular wave form potential (−0.4 to +1.3 V vs Ag/AgCl) repeated every 100 ms at a scan rate of 200 V/s (low pass Bessel filter at 3 kHz). Each cyclic voltammogram was a background-subtracted average of 10 successive cyclic voltammograms taken at the maximum oxidation peak current. All electrodes were allowed to cycle for at least 15 min before recording to stabilize the background current. The recorded current response was converted to DA concentration via *in vitro* electrode calibration with standard DA solution after each experiment. For optically evoked DA release, photostimulation during FSCV recordings was generated through a 3.4-W 447-nm LED mounted on the microscope oculars and delivered through the objective lens. Photostimulation was controlled via a DigiData 1440A, enabling control over duration and intensity. Illumination intensity typically scaled to 3 mW/mm^2^. Acquired data were analyzed and plotted using MATLAB (RRID:SCR_001622) routines and statistical analysis was performed using Prism 6.0 (GraphPad Software, RRID:SCR_002798)

### Patch-clamp electrophysiology in slices

For recordings of EPSCs and IPSCs upon optogenetic stimulation, Calb2-Cre and NEX-Cre mice (more than eight weeks, >20 g) were injected with *AAV5-EF1a-DIO-ChR2(H134R)-eYFP* as described above. Mice were deeply anaesthetized with pentobarbital (200 mg kg^−1^, i.p.; Virbac) and perfused intracardially with 10-ml ice-cold sucrose-artificial CSF (ACSF) containing: 75 mM sucrose, 87 mM NaCl, 2.5 mM KCl, 7 mM MgCl_2_, 0.5 mM CaCl_2_, 1.25 mM NaH_2_PO_4_, and 25 mM NaHCO_3_ and continuously bubbled with carbogen (95% O_2_–5% CO_2_). A total of 200-µm coronal brain slices were cut in sucrose-ACSF. Slices were transferred to a perfusion chamber containing ACSF at 31°C: 126 mM NaCl, 2.5 mM KCl, 1.2 mM MgCl_2_, 2.4 mM CaCl_2_, 1.4 mM NaH_2_PO_4_, 25 mM NaHCO_3_, and 11 mM glucose, continuously bubbled with carbogen. After at least 45-min recovery, slices were transferred to a recording chamber continuously perfused with ACSF (2–3 ml min^−1^) maintained at 29°C–31°C. Patch pipettes (3.5–5.5 MΩ) were pulled from borosilicate glass and filled with internal recording solution containing: 120 mM CsCH_3_SO_3_, 20 mM HEPES, 0.4 mM EGTA, 2.8 mM NaCl, 5 mM TEA, 2.5 mM Mg-ATP, and 0.25 mM Na-GTP, at pH 7.25 and 285 ± 5 mOsm. VTA neurons and terminals were visualized by epifluorescence and visually guided patch recordings were achieved using infrared differential interference contrast (IR-DIC) illumination (Axiocam MRm, Zeiss). ChR2 was activated by flashing blue light (5-ms pulse width) through the light path of the microscope using a light-emitting diode (UHP-LED460, Prizmatix) under computer control. EPSCs and IPSCs were recorded in whole-cell voltage clamp (–60 and 0 mV holding potential, respectively, Multiclamp 700B amplifier, Molecular Devices), filtered at 2 kHz, digitized at 10 kHz (Axon Digidata 1550, Molecular Devices), and collected online using pClamp 10 software (Molecular Device). Series resistance and capacitance were electronically compensated before recordings. Estimated liquid-junction potential was 12 mV and left uncorrected. Series resistance and/or leak current were monitored during recordings and cells that showed >25% change during recordings were considered unstable and discarded. Single-pulse (5-ms) photostimuli were applied every 55 s, and 10 photo-evoked currents were averaged per neuron per condition. DMSO stock solution of 6,7-dinitroquinoxaline-2,3-dione (DNQX; 10 mM, Sigma) was diluted 1000-fold in ACSF and bath applied. Current sizes were calculated by using peak amplitude from baseline. Decay time constants (τ) were calculated by fitting an exponential function to each averaged current trace using the following formula: f(t) = e^–t/τ^ + C.

### Place preference upon optogenetic stimulation

The three-compartment apparatus (Panlab, Harvard Apparatus) used in the CPP experiments (above) was also implemented in the optogenetics-driven place preference experiments to address RT-PP upon photostimulation and CR, the association to compartment previously paired with photostimulation. Similar to protocols previously described by others (Root et al., 2014; Qi et al., 2016), the entry of the mouse into one of the two main compartments was paired with intracranial VTA photostimulation (10-ms pulse width, 20 Hz, 10 mW) while the interconnecting compartment was not coupled to light stimulation (neutral) at all. The EthovisionXT tracking software (Noldus, RRID:SCR_000441) was used to monitor behavior and trigger laser stimulation. Behavior was assessed over the course of eight experimental days subdivided into two recording phases with a minimum 3-d rest period in between (“phase 1,” days 3–5, and “reversal phase,” days 6–8). On day 1 (“habituation”), the mouse was connected to the optic fiber cord and allowed to acclimatize. On day 2 (“pre-test”), the mouse was placed in the three-compartment apparatus for 15 min to freely explore, while attached to the optic fiber cord but without receiving any photostimulation; the preference for each compartment was evaluated. During 30 min-long recordings on days 3 and 4 (“RT-PP”), entry into the assigned light-paired compartment (non-preferred in pre-test) resulted in blue laser photostimulation delivered as continuous train of pulses (10-ms pulse width, 20 Hz, 10 mW). On day 5 (“CR”), the time spent in each compartment was measured for 15 min with no delivery of photostimulation. In the reversal phase, the protocol was repeated but with stimulation in the opposite compartment compared to phase 1. “High-power” experiments followed the same structure except that the mice received a stimulation of higher power (5-ms pulse width, 20 Hz, 20 mW).

For the Neutral Compartment Preference (NCP) test, a modified version of the test described above was used with the following changes: Entry into either one of the two main compartments was coupled to light stimulation, while only entry the interconnecting compartment had no consequence. The experiment took place on three consecutive days: During the first 2 d (Stim1 and Stim2), the mice received stimulation upon entry in any of the main compartments during 30 min long sessions while the third day was stimulation-free (15 min long session) and used to study the presence of any CRs induced by the experience with the stimulation.

### Experimental design and statistical analysis

Regular and repeated measures (RM) two-way ANOVA and unpaired *t* tests were used to compare mean scores of Ctrl and cKO mice in behavioral tests. To analyze cocaine-induced locomotion during CPP, a mixed-effects model was used. *Post hoc* comparisons were performed by Sidak’s multiple comparison test. Unpaired *t* test was used to compare mean DA release between ChR2- and eYFP (control)-injected DAT-Cre, Calb2-Cre and NEX-Cre mice for each region where the measurements were performed. Paired *t* tests were used to compare pre-DNQX and post-DNQX EPSP recordings. Two-way RM ANOVA with day and chamber were used as factors throughout the optogenetic experiments followed by Tukey’s *post hoc* test. When the days of stimulation were averaged, one-way ANOVA was used to unravel the effect of compartment (paired, unpaired, neutral) on time spent and Tukey’s multiple comparison test for *post hoc* analysis. Data are presented as mean ± SEM unless stated otherwise. Data analysis was performed with Prism8 (RRID: SCR_002798). Detailed statistical information is shown in Table 1.

**Table 1.** Statistical analysis of results obtained in behavioral and electrophysiological experiments

Figure	Data structure	Type of test	Sample Size	Statistical data
Figure 3*A* Weight analysis of ctrl and cKO mice	Normally distributed	Two-way ANOVA followed by Sidak’s multiple comparison test	Ctrl *N* = 14(M = 8, F = 6)cKO *N* = 23(M = 15, F = 8)	Interaction: *p* = 0.996, *F*_(4,158)_ = 0.0447Week: *p* < 0.001, *F*_(4,158)_ = 79.8Genotype: *p* = 0.032, *F*_(1,158)_ = 4.67Multiple comparisonsCtrl vs cKOw4 *p* = 0.908; 95% CI: –3.55 to 1.75w5 *p* = 0.966; 95% CI: –2.70 to 1.57w6 *p* = 0.876; 95% CI: –2.91 to 1.35w7 *p* = 0.720; 95% CI: –3.19 to 1.15w8 *p* = 0.783; 95%CI: –3.11 to 1.23
Figure 3*B* Baseline locomotion of ctrl and cKO mice for 30 min in 5-min bins	Normally distributed	Two-way RM ANOVA followed by Sidak’s multiple comparison test	Ctrl *N* = 17(M = 8, F = 9)cKO *N* = 17(M = 13, F = 4)	Interaction: *p* = 0.256, *F*_(5,160)_ = 1.33Time: *p <* 0.001, *F*_(5,160)_ = 69.5Genotype: *p* = 0.535, *F*_(1,32)_ = 0.00912Multiple comparisonsCtrl vs cKO5 *p >* 0.999; 95% CI: –287 to 21110 *p >* 0.999; 95% CI: –217 to 28215 *p* = 0.952; 95% CI: –170 to 32920 *p >* 0.999; 95% CI: –236 to 26325 *p* = 0.886; 95% CI: –346 to 15330 *p* = 0.993; 95% CI: –195 to 304
Figure 3*C* Sucrose preference of ctrl and cKO mice for 1%, 3%, and 10% sucrose solutions	Normally distributed	Two-way RM ANOVA followed by Sidak’s multiple comparison test	Ctrl *N* = 14(M = 8, F = 6)cKO *N* = 21(M = 13, F = 8)	Interaction: *p* = 0.475, *F*_(2,66)_ = 0.752Concentration: *p <* 0.001, *F*_(2,66)_ = 151Genotype: *p* = 0.297, *F*_(1,33)_ = 1.12Multiple comparisonsCtrl vs cKO1% *p >* 0.999; 95% CI: –5.21 to 5.693% p = 0.294; 95% CI: –1.83 to 9.0810% *p* = 0.991; 95% CI: –4.85 to 6.05
Figure 3*D* Ethanol preference of ctrl and cKO mice for 3%, 6%, and 10% ethanol concentrations	Normally distributed	Two-way RM ANOVA followed by Sidak’s multiple comparison test	Ctrl *N* = 14(M = 7, F = 7)cKO *N* = 14(M = 6, F = 8)	Interaction: *p* = 0.129, *F*_(2,52)_ = 2.13Concentration: *p <* 0.001, *F*_(2,52)_ = 14.2Genotype: *p* = 0.334, *F*_(1,26)_ = 0.969Multiple comparisonsCtrl vs cKO3% *p* = 0.983; 95% CI: –9.31 to 7.116% p = 0.453; 95% CI: –3.68 to 12.710% *p* = 0.396; 95% CI: –3.38 to 13.0Ctrl3% vs 6% *p* < 0.001; 95% CI: –14.7 to –3.453% vs 10% *p* < 0.001; 95% CI: –16.9 to –5.586% vs 10% *p* = 0.733; 95% CI: –7.78 to 3.52cKO3% vs 6% *p* = 0.354; 95% CI: –9.11 to 2.183% vs 10% *p* = 0.072; 95% CI: –10.9 to 0.3546% vs 10% *p* = 0.814; 95% CI: –7.47 to 3.82
Figure 3*E* Injection-induced locomotion for ctrl and cKO mice after saline and 5, 10, and 20 mg/kg injections of cocaine	Normally distributed	Two-way RM ANOVA followed by Sidak’s multiple comparison test	Ctrl *N* = 14(M = 8, F = 6)cKO *N* = 21(M = 13, F = 8)	Interaction: *p* = 0.396, *F*_(3,99)_ = 1Session: *p* < 0.001, *F*_(3,99)_ = 108Genotype: *p* = 0.208, *F*_(1,33)_ = 1.65Multiple comparisonsCtrl vs cKOSaline *p* = 0.966; 95% CI: –3437 to 54365 mg/kg. *p* = 0.962; 95% CI: –3410 to 546410 mg/kg. *p* = 0.887; 95% CI: –3015 to 585820 mg/kg. *p* = 0.152; 95% CI: –802 to 8071
Figure 3*F* Amphetamine- induced (3 mg/kg) locomotion under a sensitization protocol for ctrl and cKO mice	Normally distributed	Two-way RM ANOVA followed by Sidak’s multiple comparison test	Ctrl *N* = 17(M = 8, F = 9)cKO *N* = 17(M = 13, F = 4)	Interaction: *p* < 0.001, *F*_(5,160)_ = 4.79Session: *p* < 0.001, *F*_(5,160)_ = 40.9Genotype: *p* = 0.005, *F*_(1,32)_ = 9.09Multiple comparisonsCtrl vs cKODay1 *p* > 0.999; 95% CI: –13,977 to 12,091Day2 *p* = 0.266; 95% CI: –3371 to 22,696Day3 *p* = 0.063; 95% CI: –407 to 25,661Day4 *p* = 0.011; 95% CI: –2481 to 28,549Day5 *p* < 0.001; 95% CI: –6873 to 32,941Day17 *p* = 0.029; 95% CI: –928 to 26,996
Figure 3*H*Cocaine (20 mg/kg, i.p) CPP for ctrl and cKO mice	Normally distributed	Unpaired *t* test	Ctrl *N* = 12 (M = 6, F = 6)cKO *N* = 15 (M = 6, F = 9)	*t* testctrl vs cKO*p* = 0.860; 95% CI: –162.0 to 136.1
Figure 3*H*, bottom panelAmphetamine (3 mg/kg, i.p.) CPP for ctrl and cKO mice	Assumed normality	Unpaired *t* test	Ctrl *N* = 13 (M = 6, F = 7)cKO *N* = 16 (M = 9, F = 7)	*t* testctrl vs cKO*p* = 0.744; 95% CI: –365.5 to 264.3

Figure 3*I*Cocaine-induced locomotion during the CPP for ctrl and cKO mice	Assumed normality	Two-way RM ANOVA followed by Sidak’s multiple comparison test	Ctrl *N* = 12 (M = 6, F = 6)cKO *N* = 15 (M = 6, F = 9)	Interaction: *p* = 0.652, *F*_(3,75)_ = 0.5Session: *p* = 0.006, *F*_(3,75)_ = 4.4Genotype: *p* = 0.031, *F*_(1,25)_ = 5.2Multiple comparisonsCtrl vs cKOInjection 1: *p* = 0.373; 95% CI: –6850 to 1526Injection 2: *p* = 0.067; 95% CI: –8185 to 191Injection 3: *p* = 0.115; 95% CI: –7818 to 558Injection 4: *p* = 0.475; 95% CI: –6591 to 1785
Figure 3*K*, bottom panelAmphetamine-induced locomotion during the CPP for ctrl and cKO mice	Normally distributed	Mixed-effects model (REML) followed by Sidak’s multiple comparison test	Ctrl *N* = 15 (M = 7, F = 8)cKO *N* = 17 (M = 10, F = 7)	Interaction: *p* = 0.567, *F*_(3,85)_ = 0.680Session: *p* < 0.001, *F*_(3,85)_ = 24.0Genotype: *p* = 0.803, *F*_(1,30)_ = 0.0631Multiple comparisonsCtrl vs cKOInjection 1: *p* = 0.941; 95% CI: –2522 to 1473Injection 2: *p* = 0.931; 95% CI: –1431 to 2517Injection 3: *p* = 0.995; 95% CI: –1783 to 2331Injection 4: *p* = 0.989; 95% CI: –1671 to 2327
Figure 6*D*, leftOptically evoked DA release in NAcSh of DAT-, NEX-, and Calb2-Cre mice injected with ChR2 or eYFP	Normally distributed	Unpaired *t* test	10 observations for each group and virusDAT-Cre/Chr2 *N* = 2(M = 0, F = 2)DAT-Cre/eYFP *N* = 2(M = 1, F = 1)NEX-Cre/Chr2 *N* = 3(M = 2, F = 1)NEX-Cre/eYFP *N* = 2(M = 0, F = 2)Calb2-Cre/Chr2 *N* = 3(M = 1, F = 2)Calb2-Cre/eYFP *N* = 2(M = 0, F = 2)	*t* testDAT-Cre/ChR2 vs DAT-Cre/eYFP*p* < 0.0001; 95% CI: –1.272 to –0.6540NEX-Cre/ChR2 vs NEX-Cre/eYFP*p* < 0.0001; 95% CI: –0.6289 to –0.2909Calb2-Cre/ChR2 vs Calb2-Cre/eYFP*p* = 0.0148; 95% CI: –0.01602 to –0.001988
Figure 6*D*, rightOptically evoked DA release in OT of DAT-, NEX-, and Calb2-Cre mice injected with ChR2 or eYFP	Normally distributed	Unpaired *t* test	As above	*t* testDAT-Cre/ChR2 vs DAT-Cre/eYFP*p* < 0.0001; 95% CI: –0.2354 to –0.1810NEX-Cre/ChR2 vs NEX-Cre/eYFP*p* = 0.0049; 95% CI: –0.01295 to –0.002704Calb2-Cre/ChR2 vs Calb2-Cre/eYFP*p* = 0.0002; 95% CI: –0.02022 to –0.007554
Figure 7*D*, left panelOptically evoked EPSCs in NAcSh of NEX-Cre/ChR2 mice before (control) and after DNQX bath application	Assumed normality	Paired *t* test	6 cells from 3 NEX-Cre/ChR2 mice(M = 3, F = 0)	*p* = 0.0481; 95% CI: –61.86 to –0.3739
Figure 7*D*, right panelOptically evoked EPSCs in OT of Calb2-Cre/ChR2 mice before (control) and after DNQX bath application	Assumed normality	Paired *t* test	5 cells from 3 Calb2-Cre/ChR2 mice(M = 2, F = 1)	*p* = 0.0456; 95% CI: –89.88 to –1.444
Figure 8*B*, leftBehavioral analysis of DAT-Cre/ChR2 mice throughout the opto-behavioral experiments	Normally distributed	Two-way RM ANOVA followed by Tukey’s multiple comparison test	*N* = 10(M = 2, F = 8)	Interaction: *p* < 0.001, *F*_(12,108)_ = 33Day: *p* = 0.435, *F*_(6,54)_ = 1Compartment: *p* < 0.001, *F*_(2,18)_ = 51.8Multiple comparisons (of interest)Day2 (pre-test)Paired vs unpaired *p* = 0.513; 95% CI: –38.4 to –6.29Day3 (RT-PP)Paired vs unpaired *p <* 0.001; 95% CI: 21.7 to 66.4Paired vs neutral *p <* 0.001; 95% CI: 35.7 to 80.4Day4 (RT-PP)Paired vs unpaired *p <* 0.001; 95% CI: 37.6 to 82.3Paired vs neutral *p <* 0.001; 95% CI: 47.3 to 92.0Day5 (CR)Paired vs unpaired *p <* 0.001; 95% CI: 11.5 to 56.1Paired vs neutral *p <* 0.001; 95% CI: 26.7 to 71.4Day6 (RT-PP)Paired vs unpaired *p <* 0.001; 95% CI: –65.7 to –21.0Paired vs neutral *p <* 0.001; 95% CI: 34.4 to 79.1Day7 (RT-PP)Paired vs unpaired *p <* 0.001; 95% CI: –85.0 to –40.4Paired vs neutral *p <* 0.001; 95% CI: 43.1 to 87.7Day8 (CR)Paired vs unpaired *p <* 0.001; 95% CI: –67.2 to –22.5Paired vs neutral *p <* 0.001; 95% CI: 33.6 to 78.3

				Reversal parameters:Day3 paired vs Day6 unpaired *p <* 0.001; 95% CI: 22.8 to 67.4Day3 paired vs Day7 unpaired *p <* 0.001; 95% CI: 32.5 to 77.2Day4 paired vs Day6 unpaired *p <* 0.001; 95% CI: 32.3 to 76.9Day4 paired vs Day7 unpaired *p <* 0.001; 95% CI: 42.0 to 86.7Day5 paired vs Day8 unpaired *p <* 0.001; 95% CI: 17.5 to 62.2
Figure 8*B*, rightTime spent in paired, unpaired, and neutral compartments during the four RT-PP days for DAT-Cre/ChR2 mice	Normally distributed	RM one-way ANOVA followed by Tukey’s multiple comparison test	*N* = 10(M = 2, F = 8)	Compartment *p* < 0.001, *F*_(2,6)_ = 166Multiple comparisonsPaired vs unpaired *p <* 0.001; 95% CI: 41.1 to 63.9Paired vs neutral *p <* 0.001; 95% CI: 51.7 to 74.5Unpaired vs neutral *p =* 0.066; 95% CI: –0.808 to 22.0
Extended Data Figure 8-1*A*, leftBehavioral analysis of DAT-Cre-negative mice injected with AAV-ChR2 throughout the opto-behavioral experiments	Normally distributed	Two-way RM ANOVA followed by Tukey’s multiple comparison test	*N* = 3(M = 0, F = 3)	Interaction: *p* = 0.562, *F*_(12,24)_ = 0.898Day: *p* = 0.569, *F*_(6,12)_ = 0.830Compartment: *p* = 0.102, *F*_(2,4)_ = 4.26Multiple comparisons (of interest)Day2 (pre-test)Paired vs unpaired *p* = 0.010; 95% CI: –49.6 to –4.05Day3 (RT-PP)Paired vs unpaired *p* = 0.074; 95% CI: 44.5 to 1.05Paired vs neutral *p* = 0.292; 95% CI: –5.3 to 40.3Day4 (RT-PP)Paired vs unpaired *p* = 0.236; 95% CI: –41.0 to 4.57Paired vs neutral *p* = 0.055; 95% CI: –0.241 to 45.3Day5 (CR)Paired vs unpaired *p* = 0.074; 95% CI: –44.5 to 1.08Paired vs neutral *p* = 0.204; 95% CI: –4.08 to 41. 5Day6 (RT-PP)Paired vs unpaired *p* = 0.998; 95% CI: –30.3 to 15.3Paired vs neutral *p <* 0.001; 95% CI: 12.0 to 57.6Day7 (RT-PP)Paired vs unpaired *p* = 0.863; 95% CI: –34.5 to 11.0Paired vs neutral *p* = 0.001; 95% CI: 9.18 to 54.8Day8 (CR)Paired vs unpaired *p* = 0.012; 95% CI: –49.2 to –3.6Paired vs neutral *p <* 0.001; 95% CI: 22.5 to 68.1Reversal parameters:Day3 paired vs Day6 unpaired *p* = 0.995; 95% CI: –30.8 to 14.8Day3 paired vs Day7 unpaired *p >* 0.999; 95% CI: –27.0 to 18.5Day4 paired vs Day6 unpaired *p >* 0.999; 95% CI: –28.0 to 17.6Day4 paired vs Day7 unpaired *p >* 0.999; 95% CI: –24.2 to 21.4Day5 paired vs Day8 unpaired *p >* 0.999; 95% CI: –21.3 to 24.3
Extended Data Figure 8-1*A*, rightTime spent in paired, unpaired, and neutral compartments during the four RT-PP days for DAT-Cre-negative/ChR2 mice	Normally distributed	RM one-way ANOVA followed by Tukey’s multiple comparison test	*N* = 3(M = 0, F = 3)	Compartment *p* < 0.001, *F*_(2,6)_ = 48.7Multiple comparisonsPaired vs unpaired *p =* 0.358; 95% CI: –15.8 to 5.46Paired vs neutral *p <* 0.001; 95% CI: 16.1 to 37.3Unpaired vs neutral *p <* 0.001; 95% CI: 21.2 to 42.5
Extended Data Figure 8-1*B*, leftBehavioral analysis of DAT-Cre/eYFP throughout the opto-behavioral experiments	Normally distributed	Two-way RM ANOVA followed by Tukey’s multiple comparison test	*N* = 3(M = 0, F = 3)	Interaction: *p* = 0.677, *F*_(12,24)_ = 0.767Day: *p* = 0.935, *F*_(6,12)_ = 0.281Compartment: *p* = 0.004, *F*_(2,4)_ = 27.9Multiple comparisons (of interest)Day2 (pre-test)Paired vs unpaired *p* < 0.001; 95% CI: –62.8 to –22.5Day3 (RT-PP)Paired vs unpaired *p* < 0.001; 95% CI: –64.9 to –24.6Paired vs neutral *p* = 0.198; 95% CI: –3.52 to 36.7Day4 (RT-PP)Paired vs unpaired *p* < 0.001; 95% CI: –63.5 to –23.2Paired vs neutral *p* = 0.222; 95% CI: –3.85 to 36.4Day5 (CR)Paired vs unpaired *p* < 0.001; 95% CI: –65.9 to –25.6Paired vs neutral *p* = 0.251; 95% CI: –4.21 to 36.1Day6 (RT-PP)Paired vs unpaired *p* < 0.001; 95% CI: –70.7 to –30.5Paired vs neutral *p <* 0.001; 95% CI: 45.8 to 86.1Day7 (RT-PP)Paired vs unpaired *p* < 0.001; 95% CI: –55.5 to –15.2Paired vs neutral *p* < 0.001; 95% CI: 31.3 to 71.6Day8 (CR)Paired vs unpaired *p* < 0.001; 95% CI: –66.1 to –25.8Paired vs neutral *p* < 0.001; 95% CI: –42.7 to 82.9

				Reversal parameters:Day3 paired vs Day6 unpaired *p* > 0.999; 95% CI: –17.8 to 22.5Day3 paired vs Day7 unpaired *p >* 0.999; 95% CI: –23.1 to 17.2Day4 paired vs Day6 unpaired *p >* 0.999; 95% CI: –17.4 to 22.9Day4 paired vs Day7 unpaired *p >* 0.999; 95% CI: –22.7 to 17.5Day5 paired vs Day8 unpaired *p >* 0.999; 95% CI: –20.4 to 19.9
Extended Data Figure 8-1*B*, rightTime spent in paired, unpaired, and neutral compartments during the four RT-PP days for DAT-Cre/eYFP mice	Normally distributed	RM one-way ANOVA followed by Tukey’s multiple comparison test	*N* = 3(M = 0, F = 3)	Compartment *p* = 0.127, *F*_(2,6)_ = 2.97Multiple comparisonsPaired vs unpaired *p >* 0.999; 95% CI: –55.6 to 54.5Paired vs neutral *p =* 0.171; 95% CI: –17.5 to 92.6Unpaired vs neutral *p =* 0.165; 95% CI: –16.9 to 93.1
Extended Data Figure 8-1*C*, leftBehavioral analysis of DAT-Cre controls (pooled) throughout the opto-behavioral experiments	Normally distributed	Two-way RM ANOVA followed by Tukey’s multiple comparison test	*N* = 6(M = 0, F = 6)	Interaction: *p* = 0.494, *F*_(12,60)_ = 0.963Day: *p* = 0.929, *F*_(6,30)_ = 0.306Compartment: *p* < 0.001 *F*_(2,10)_ = 18.6Multiple comparisons (of interest)Day2 (pre-test)Paired vs unpaired *p* < 0.001; 95% CI: –48.6 to –20.8Day3 (RT-PP)Paired vs unpaired *p* < 0.001; 95% CI: –47.1 to –19.4Paired vs neutral *p* = 0.004; 95% CI: 3.15 to 30.9Day4 (RT-PP)Paired vs unpaired *p* < 0.001; 95% CI: –44.7 to –16.9Paired vs neutral *p* < 0.001; 95% CI: 5.52 to 33.3Day5 (CR)Paired vs unpaired *p* < 0.001; 95% CI: –47.6 to –19.8Paired vs neutral *p* = 0.003; 95% CI: 3.42 to 31.2Day6 (RT-PP)Paired vs unpaired *p* < 0.001; 95% CI: –42.9 to –15.1Paired vs neutral *p <* 0.001; 95% CI: 36.5 to 64.3Day7 (RT-PP)Paired vs unpaired *p* < 0.001; 95% CI: –37.4 to –9.64Paired vs neutral *p <* 0.001; 95% CI: 27.8 to 55.6Day8 (CR)Paired vs unpaired *p* < 0.001; 95% CI: –50.1 to –22.3Paired vs neutral *p* < 0.001; 95% CI: 40.2 to 67.9Reversal parameters:Day3 paired vs Day6 unpaired *p* > 0.999; 95% CI: –16.7 to 11.1Day3 paired vs Day7 unpaired *p >* 0.999; 95% CI: –17.5 to 10.3Day4 paired vs Day6 unpaired *p >* 0.999; 95% CI: –15.1 to 12.7Day4 paired vs Day7 unpaired *p >* 0.999; 95% CI: –15.9 to 11.9Day5 paired vs Day8 unpaired *p >* 0.999; 95% CI: –13.3 to 14.5
Extended Data Figure 8-1*C*, rightTime spent in paired, unpaired, and neutral compartments during the four RT-PP days for DAT-Cre control mice (pooled)	Normally distributed	RM one-way ANOVA followed by Tukey’s multiple comparison test	*N* = 6(M = 0, F = 6)	Compartment *p* = 0.015, *F*_(2,6)_ = 9.27Multiple comparisonsPaired vs unpaired *p =* 0.946; 95% CI: –30.6 to 24.8Paired vs neutral *p =* 0.028; 95% CI: 4.44 to 59.8Unpaired vs neutral *p =* 0.019; 95% CI: 7.30 to 62.7
Extended Data Figure 8-1*E*, leftBehavioral analysis of DAT-Cre/ChR2 mice tested on high power, throughout the opto-behavioral experiments	Normally distributed	Two-way RM ANOVA followed by Tukey’s multiple comparison test	*N* = 10(M = 2, F = 8)	Interaction: *p* < 0.001, *F*_(12,36)_ = 22.6Day: *p* = 0.455, *F*_(6,18)_ = 1Compartment: *p* < 0.001, *F*_(2,6)_ = 105Multiple comparisons (of interest)Day2 (pre-test)Paired vs unpaired *p* > 0.999; 95% CI: –45.7 to 26.7Day3 (RT-PP)Paired vs unpaired *p <* 0.001; 95% CI: 26.2 to 98.6Paired vs neutral *p <* 0.001; 95% CI: 38.5 to 111Day4 (RT-PP)Paired vs unpaired *p <* 0.001; 95% CI: 42.7 to 115Paired vs neutral *p <* 0.001; 95% CI: 47.1 to 119Day5 (CR)Paired vs unpaired *p <* 0.001; 95% CI: 17.4 to 89.8Paired vs neutral *p <* 0.001; 95% CI: 16.5 to 88.8Day6 (RT-PP)Paired vs unpaired *p <* 0.001; 95% CI: –98.0 to –25.6Paired vs neutral *p <* 0.001; 95% CI: 36.5 to 109Day7 (RT-PP)Paired vs unpaired *p <* 0.001; 95% CI: –109 to –37.0Paired vs neutral *p <* 0.001; 95% CI: 42.3 to 115Day8 (CR)Paired vs unpaired *p =* 0.407; 95% CI: –62.5 to 9.72Paired vs neutral *p =* 0.030; 95% CI: 1.98 to 74.3

				Reversal parameters:Day3 paired vs Day6 unpaired *p <* 0.001; 95% CI: 26.5 to 98.9Day3 paired vs Day7 unpaired *p <* 0.001; 95% CI: 32.1 to 104Day4 paired vs Day6 unpaired *p <* 0.001; 95% CI: 34.8 to 107Day4 paired vs Day7 unpaired *p <* 0.001; 95% CI: 40.5 to 113Day5 paired vs Day8 unpaired *p <* 0.001; 95% CI: 4.15 to 76.5
Extended Data Figure 8-1*E*, rightTime spent in paired, unpaired, and neutral compartments during the four RT-PP days for DAT-Cre/ChR2 mice under high power stimulation	Normally distributed	RM one-way ANOVA followed by Tukey’s multiple comparison test	*N* = 4(M = 0, F = 4)	Compartment *p* < 0.001, *F*_(2,6)_ = 404Multiple comparisonsPaired vs unpaired *p <* 0.001; 95% CI: 59.9 to 78.2Paired vs neutral *p <* 0.001; 95% CI: 68.1 to 86.5Unpaired vs neutral *p =* 0.074; 95% CI: –0.934 to 17.4
Figure 8*C*, leftBehavioral analysis of Vglut2-Cre/ChR2 mice throughout the opto-behavioral experiments	Normally distributed	Two-way RM ANOVA followed by Tukey’s multiple comparison test	*N* = 7(M = 2, F = 5)	Interaction: *p* < 0.001, *F*_(12,72)_ = 16.1Day: *p* = 0.181, *F*_(6,36)_ = 1.58Compartment: *p* < 0.001, *F*_(2,12)_ = 40.9Multiple comparisons (of interest)Day2 (pre-test)Paired vs unpaired *p* > 0.999; 95% CI: –28.1 to 26.6Day3 (RT-PP)Paired vs unpaired *p <* 0.001; 95% CI: –71.9 to –17.2Paired vs neutral *p* > 0.999; 95% CI: –18.0 to 36.7Day4 (RT-PP)Paired vs unpaired *p <* 0.001; 95% CI: –68.1 to –13.4Paired vs neutral *p* > 0.997; 95% CI: –16.9 to 37.8Day5 (CR)Paired vs unpaired *p* = 0.998; 95% CI: –37.8 to 16.9Paired vs neutral *p* = 0.019; 95% CI: 2.42 to 57.1Day6 (RT-PP)Paired vs unpaired *p <* 0.001; 95% CI: 32.9 to 87.6Paired vs neutral *p* > 0.999; 95% CI: –28.8 to 25.9Day7 (RT-PP)Paired vs unpaired *p <* 0.001; 95% CI: 27.0 to 81.7Paired vs neutral *p* > 0.999; 95% CI: –28.9 to 25.8Day8 (CR)Paired vs unpaired *p* = 0.783; 95% CI: –10.8 to 43.9Paired vs neutral *p* = 0.055; 95% CI: –0.268 to 54.4Reversal parameters:Day3 paired vs Day6 unpaired *p <* 0.001; 95% CI: –78.7 to –24.1Day3 paired vs Day7 unpaired *p <* 0.001; 95% CI: –74.8 to –20.1Day4 paired vs Day6 unpaired *p <* 0.001; 95% CI: –77.1 to –22.4Day4 paired vs Day7 unpaired *p <* 0.001; 95% CI: –73.2 to –18.5Day5 paired vs Day8 unpaired *p* = 0.952; 95% CI: –41.0 to 13.7
Figure 8*C* (right) time spent in paired, unpaired and neutral compartments during the 4 RT-PP days for Vglut2-Cre/ChR2 mice	Normally distributed	RM one-way ANOVA followed by Tukey’s multiple comparison test	*N* = 7(M = 2, F = 5)	Compartment *p* < 0.001, *F*_(2,6)_ = 162Multiple comparisonsPaired vs unpaired *p <* 0.001; 95% CI: –60.2 to –39.7Paired vs neutral *p =* 0.469; 95% CI: –6.08 to 14.5Unpaired vs neutral *p <* 0.001; 95% CI: 43.9 to 64.4
Figure 8*D*, leftBehavioral analysis of Calb2-Cre/ChR2 mice throughout the opto-behavioral experiments	Normally distributed	Two-way RM ANOVA followed by Tukey’s multiple comparison test	*N* = 7(M = 0, F = 7)	Interaction: *p* = 0.163, *F*_(12,72)_ = 1.45Day: *p* = 0.567, *F*_(6,36)_ = 0.813Compartment: *p* < 0.001, *F*_(2,12)_ = 27Multiple comparisons (of interest)Day2 (pre-test)Paired vs unpaired *p* = 0.096; 95% CI: –33.3 to 1.13Day3 (RT-PP)Paired vs unpaired *p =* 0.343; 95% CI: –30.6 to 3.82Paired vs neutral *p* = 0.010; 95% CI: 2.52 to 37.0Day4 (RT-PP)Paired vs unpaired *p* > 0.999; 95% CI: –21.1 to 13.4Paired vs neutral *p <* 0.001; 95% CI: 8.22 to 42.7Day5 (CR)Paired vs unpaired *p* > 0.999; 95% CI: 13.4 to 47.9Paired vs neutral *p <* 0.001; 95% CI: –15.7 to 18.8Day6 (RT-PP)Paired vs unpaired *p* > 0.999; 95% CI: –18.2 to 16.2Paired vs neutral *p <* 0.001; 95% CI: 13.0 to 47.5Day7 (RT-PP)Paired vs unpaired *p <* 0.001; 95% CI: –17.6 to 16.9Paired vs neutral *p* > 0.999; 95% CI: 10.8 to 45.2Day8 (CR)Paired vs unpaired *p <* 0.001; 95% CI: –13.1 to 21.3Paired vs neutral *p* > 0.999; 95% CI: 7.45 to 41.9

				Reversal parameters:Day3 paired vs Day6 unpaired *p =* 0.991; 95% CI: –24.6 to 9.91Day3 paired vs Day7 unpaired *p =* 0.995; 95% CI: –24.2 to 10.2Day4 paired vs Day6 unpaired *p* > 0.999; 95% CI: –19.5 to 15.0Day4 paired vs Day7 unpaired *p* > 0.999; 95% CI: –19.1 to 15.3Day5 paired vs Day8 unpaired *p* > 0.999; 95% CI: –17.2 to 17.3
Figure 8*D*, rightTime spent in paired, unpaired, and neutral compartments during the four RT-PP days for Calb2-Cre/ChR2 mice	Normally distributed	RM one-way ANOVA followed by Tukey’s multiple comparison test	*N* = 7(M = 0, F = 7)	Compartment*p* < 0.001, *F*_(2,6)_ = 90.1Multiple comparisonsPaired vs unpaired *p =* 0.297; 95% CI: –11.4 to 3.42Paired vs neutral *p <* 0.001; 95% CI: 18.5 to 33.3Unpaired vs neutral *p <* 0.001; 95% CI: 22.4 to 37.3
Extended Data Figure 8-1*F*Behavioral analysis of Calb2-Cre/ChR2 mice tested on high power, throughout the opto-behavioral experiments	Normally distributed	Two-way RM ANOVA followed by Tukey’s multiple comparison test	*N* = 7(M = 0, F = 7)	Interaction: *p* = 0.927, *F*_(12,72)_ = 0.469Day: *p* = 0.661, *F*_(6,36)_ = 0.688Compartment: *p* = 0.001, *F*_(2,12)_ = 12.5Multiple comparisons (of interest)Day2 (pre-test)Paired vs unpaired *p* = 0.104; 95% CI: –33.5 to 1.28Day3 (RT-PP)Paired vs unpaired *p =* 0.995; 95% CI: –24.4 to 10.4Paired vs neutral *p =* 0.019; 95% CI: 1.56 to 36.3Day4 (RT-PP)Paired vs unpaired *p >* 0.999; 95% CI: –22.6 to 12.2Paired vs neutral *p <* 0.001; 95% CI: 47.1 to 119Day5 (CR)Paired vs unpaired *p =* 0.742; 95% CI: –28.2 to 6.54Paired vs neutral *p =* 0.015; 95% CI: 1.87 to 36.6Day6 (RT-PP)Paired vs unpaired *p =* 0.937; 95% CI: –26.3 to 8.46Paired vs neutral *p <* 0.001; 95% CI: 11.1 to 45.9Day7 (RT-PP)Paired vs unpaired *p >* 0.999; 95% CI: –22.3 to 12.5Paired vs neutral *p <* 0.001; 95% CI: 7.32 to 42.1Day8 (CR)Paired vs unpaired *p =* 0.976; 95% CI: –25.5 to 9.30Paired vs neutral *p <* 0.001; 95% CI: 7.52 to 42.3Reversal parameters:Day3 paired vs Day6 unpaired *p >* 0.999; 95% CI: –17.0 to 17.8Day3 paired vs Day7 unpaired *p >* 0.999; 95% CI: –18.4 to 16.4Day4 paired vs Day6 unpaired *p >* 0.999; 95% CI: –15.1 to 19.6Day4 paired vs Day7 unpaired *p >* 0.999; 95% CI: –16.5 to 18.2Day5 paired vs Day8 unpaired *p >* 0.999; 95% CI: –17.5 to 17.3
Extended Data Figure 8-1*F*, rightTime spent in paired, unpaired, and neutral compartments during the four RT-PP days for Calb2-Cre/ChR2 mice under high power stimulation	Normally distributed	RM one-way ANOVA followed by Tukey’s multiple comparison test	*N* = 7(M = 0, F = 7)	Compartment *p* < 0.001, *F*_(2,6)_ = 47.3Multiple comparisonsPaired vs unpaired *p =* 0.988; 95% CI: –8.15 to 8.97Paired vs neutral *p <* 0.001; 95% CI: 15.1 to 32.3Unpaired vs neutral *p <* 0.001; 95% CI: 14.7 to 31.8
Figure 8*E*, leftBehavioral analysis of NEX-Cre/ChR2 mice throughout the opto-behavioral experiments	Normally distributed	Two-way RM ANOVA followed by Tukey’s multiple comparison test	*N* = 5(M = 1, F = 4)	Interaction: *p* < 0.001, *F*_(12,48)_ = 4.63Day: *p* = 0.307, *F*_(6,24)_ = 1.27Compartment: *p* < 0.001, *F*_(2,8)_ = 76.8Multiple comparisons (of interest)Day2 (pre-test)Paired vs unpaired *p* > 0.999; 95% CI: –18.7 to 24.9Day3 (RT-PP)Paired vs unpaired *p =* 0.414; 95% CI: –5.70 to 37.9Paired vs neutral *p <* 0.001; 95% CI: 17.1 to 60.7Day4 (RT-PP)Paired vs unpaired *p* > 0.999; 95% CI: –17.5 to 26.1Paired vs neutral *p <* 0.001; 95% CI: 16.6 to 60.3Day5 (CR)Paired vs unpaired *p* > 0.999; 95% CI: 12.0 to 55.6Paired vs neutral *p <* 0.001; 95% CI: –5.03 to 38.6Day6 (RT-PP)Paired vs unpaired *p* = 0.020; 95% CI: –45.5 to –1.92Paired vs neutral *p <* 0.001; 95% CI: 15.1 to 58.7Day7 (RT-PP)Paired vs unpaired *p <* 0.001; 95% CI: –51.8 to –8.16Paired vs neutral *p <* 0.001; 95% CI: 20.5 to 64.1Day8 (CR)Paired vs unpaired *p =* 0.937; 95% CI: –32.7 to 10.9Paired vs neutral *p <* 0.001; 95% CI: 16.3 to 59.9

				Reversal parameters:Day3 paired vs Day6 unpaired *p =* 0.049; 95% CI: 0.0239 to 43.6Day3 paired vs Day7 unpaired *p =* 0.016; 95% CI: 2.38 to 46.0Day4 paired vs Day6 unpaired *p =* 0.252; 95% CI: –4.06 to 39.5Day4 paired vs Day7 unpaired *p =* 0.105; 95% CI: –1.71 to 41.9Day5 paired vs Day8 unpaired *p =* 0.998; 95% CI: –14.0 to 29.6
Figure 8*E*, rightTime spent in paired, unpaired, and neutral compartments during the four RT-PP days for NEX-Cre/ChR2 mice	Normally distributed	RM one-way ANOVA followed by Tukey’s multiple comparison test	*N* = 5(M = 1, F = 4)	Compartment *p* < 0.001, *F*_(2,6)_ = 39.7Multiple comparisonsPaired vs unpaired *p =* 0.013; 95% CI: 5.03 to 32.0Paired vs neutral *p <* 0.001; 95% CI: 25.7 to 52.6Unpaired vs neutral *p =* 0.008; 95% CI: 7.16 to 34.1
Extended Data Figure 8-1*G*, leftBehavioral analysis of NEX-Cre/ChR2 mice tested on high power, throughout the opto-behavioral experiments	Normally distributed	Two-way RM ANOVA followed by Tukey’s multiple comparison test	*N* = 4(M = 1, F = 3)	Interaction: *p* < 0.001, *F*_(12,36)_ = 8.58Day: *p* = 0.252, *F*_(6,18)_ = 1.44Compartment: *p* < 0.001, *F*_(2,6)_ = 48.3Multiple comparisons (of interest)Day2 (pre-test)Paired vs unpaired *p* = 0.369; 95% CI: –25.0 to 3.62Day3 (RT-PP)Paired vs unpaired *p =* 0.358; 95% CI: –3.54 to 25.1Paired vs neutral *p <* 0.001; 95% CI: 16.0 to 44.6Day4 (RT-PP)Paired vs unpaired *p =* 0.003; 95% CI: 3.97 to 32.6Paired vs neutral *p <* 0.001; 95% CI: 24.9 to 53.5Day5 (CR)Paired vs unpaired *p =* 0.084; 95% CI: –0.819 to 27.8Paired vs neutral *p <* 0.001; 95% CI: 19.7 to 48.3Day6 (RT-PP)Paired vs unpaired *p =* 0.087; 95% CI: –27.8 to 0.877Paired vs neutral *p <* 0.001; 95% CI: 17.7 to 46.4Day7 (RT-PP)Paired vs unpaired *p <* 0.001; 95% CI: –34.7 to –6.03Paired vs neutral *p <* 0.001; 95% CI: 21.8 to 50.5Day8 (CR)Paired vs unpaired *p =* 0.798; 95% CI: –22.5 to 6.11Paired vs neutral *p <* 0.001; 95% CI: 13.7 to 42.3Reversal parameters:Day3 paired vs Day6 unpaired *p =* 0.203; 95% CI: –2.36 to 26.3Day3 paired vs Day7 unpaired *p =* 0.028; 95% CI: 0.881 to 29.5Day4 paired vs Day6 unpaired *p =* 0.005; 95% CI: 3.12 to 31.8Day4 paired vs Day7 unpaired *p <* 0.001; 95% CI: 6.36 to 35.0Day5 paired vs Day8 unpaired *p =* 0.202; 95% CI: –2.35 to 26.3
Extended Data Figure 8-1*G*, rightTime spent in paired, unpaired, and neutral compartments during the four RT-PP days for NEX-Cre/ChR2 mice under high-power stimulation	Normally distributed	RM one-way ANOVA followed by Tukey’s multiple comparison test	*N* = 4(M = 1, F = 3)	Compartment *p* < 0.001, *F*_(2,6)_ = 178Multiple comparisonsPaired vs unpaired *p <* 0.001; 95% CI: 10.1 to 21.3Paired vs neutral *p <* 0.001; 95% CI: 28.8 to 40.0Unpaired vs neutral *p <* 0.001; 95% CI: 13.1 to 24.3
Extended Data Figure 8-1*H*, leftBehavioral analysis of bilaterally injected NEX-Cre/ChR2 mice throughout the opto-behavioral experiments	Normally distributed	Two-way RM ANOVA followed by Tukey’s multiple comparison test	*N* = 4(M = 0, F = 4)	Interaction: *p* = 0.040, *F*_(12,36)_ = 2.13Day: *p* = 0.384, *F*_(6,18)_ = 1.13Compartment: *p* < 0.001, *F*_(2,6)_ = 43.3Multiple comparisons (of interest)Day2 (pre-test)Paired vs unpaired *p* = 0.999; 95% CI: –50.7 to 25.1Day3 (RT-PP)Paired vs unpaired *p =* 0.998; 95% CI: –24.6 to 51.2Paired vs neutral *p =* 0.017; 95% CI: 4.27 to 80.1Day4 (RT-PP)Paired vs unpaired *p =* 0.768; 95% CI: –15.7 to 60.2Paired vs neutral *p =* 0.003; 95% CI: 10.3 to 86.1Day5 (CR)Paired vs unpaired *p =* 0.974; 95% CI: –21.2 to 54.7Paired vs neutral *p =* 0.015; 95% CI: 4.63 to 80.5Day6 (RT-PP)Paired vs unpaired *p >* 0.999; 95% CI: –49.6 to 26.3Paired vs neutral *p =* 0.029; 95% CI: 2.13 to 78.0Day7 (RT-PP)Paired vs unpaired *p =* 0.999; 95% CI: –52.5 to 23.3Paired vs neutral *p =* 0.019; 95% CI: 3.69 to 79.5Day8 (CR)Paired vs unpaired *p =* 0.185; 95% CI: –70.1 to 5.78Paired vs neutral *p <* 0.001; 95% CI: 14.2 to 90.0

				Reversal parameters:Day3 paired vs Day6 unpaired *p =* 0.999; 95% CI: –25.0 to 50.8Day3 paired vs Day7 unpaired *p =* 0.995; 95% CI: –23.6 to 52.3Day4 paired vs Day6 unpaired *p =* 0.952; 95% CI: –20.0 to 55.8Day4 paired vs Day7 unpaired *p =* 0.999; 95% CI: –18.6 to 57.3Day5 paired vs Day8 unpaired *p =* 0.668; 95% CI: –14.1 to 61.8
Extended Data Figure 8-1*H*, rightTime spent in paired, unpaired, and neutral compartments during the four RT-PP days for bilaterally injected NEX-Cre/ChR2 mice	Normally distributed	RM one-way ANOVA followed by Tukey’s multiple comparison test	*N* = 4(M = 0, F = 4)	Compartment *p* < 0.001, *F*_(2,6)_ = 331Multiple comparisonsPaired vs unpaired *p <* 0.001; 95% CI: 10.1 to 20.8Paired vs neutral *p <* 0.001; 95% CI: 37.7 to 48.4Unpaired vs neutral *p <* 0.001; 95% CI: 22.2 to 32.9
Figure 8*F*, left Behavioral analysis of bilaterally injected NEX-Cre/ChR2 mice throughout the opto-behavioral experiments, tested on high power	Normally distributed	Two-way RM ANOVA followed by Tukey’s multiple comparison test	*N* = 4(M = 0, F = 4)	Interaction: *p* < 0.001, *F*_(12,36)_ = 9.03Day: *p* = 0.310, *F*_(6,18)_ = 1.29Compartment: *p* < 0.001, *F*_(2,6)_ = 36.5Multiple comparisons (of interest)Day2 (pre-test)Paired vs unpaired *p* = 0.982; 95% CI: –42.9 to 17.4Day3 (RT-PP)Paired vs unpaired *p =* 0.349; 95% CI: –7.34 to 53.0Paired vs neutral *p <* 0.001; 95% CI: 20.0 to 80.3Day4 (RT-PP)Paired vs unpaired *p <* 0.001; 95% CI: 14.1 to 74.4Paired vs neutral *p <* 0.001; 95% CI: 30.8 to 91.1Day5 (CR)Paired vs unpaired *p >* 0.999; 95% CI: –29.7 to 30.6Paired vs neutral *p =* 0.002; 95% CI: 9.24 to 69.5Day6 (RT-PP)Paired vs unpaired *p <* 0.001; 95% CI: –76.1 to –15.8Paired vs neutral *p <* 0.001; 95% CI: 28.5 to 88.8Day7 (RT-PP)Paired vs unpaired *p <* 0.001; 95% CI: –74.4 to –14.1Paired vs neutral *p <* 0.001; 95% CI: 27.0 to 87.3Day8 (CR)Paired vs unpaired *p =* 0.989; 95% CI: –42.4 to 17.9Paired vs neutral *p =* 0.006; 95% CI: 6.19 to 66.5Reversal parameters:Day3 paired vs Day6 unpaired *p =* 0.009; 95% CI: 5.24 to 65.5Day3 paired vs Day7 unpaired *p =* 0.011; 95% CI: 4.65 to 64.9Day4 paired vs Day6 unpaired *p <* 0.001; 95% CI: 16.0 to 76.3Day4 paired vs Day7 unpaired *p <* 0.001; 95% CI: 15.4 to 75.7Day5 paired vs Day8 unpaired *p >* 0.999; 95% CI: –20.8 to 39.5
Figure 8*F*, rightTime spent in paired, unpaired, and neutral compartments during the four RT-PP days for NEX-Cre/ChR2 mice bilaterally injected and under high-power stimulation	Assumed normality	RM one-way ANOVA followed by Tukey’s multiple comparison test	*N* = 4(M = 0, F = 4)	Compartment *p* < 0.001, *F*_(2,6)_ = 106Multiple comparisonsPaired vs unpaired *p <* 0.001; 95% CI: 27.1 to 51.6Paired vs neutral *p <* 0.001; 95% CI: 44.5 to 69.0Unpaired vs neutral *p =* 0.011; 95% CI: 5.19 to 29.7
Extended Data Figure 8-1*Iii*Behavioral analysis of Vglut2-Cre throughout the NCP experiments	Normally distributed	RM one-way ANOVA followed by Tukey’s multiple comparison test	*N* = 5(M = 0, F = 5)	Interaction: *p* = 0.002, *F*_(4,16)_ = 6.90Day: *p* = 0.410, *F*_(2,8)_ = 1Compartment: *p* < 0.001, *F*_(2,8)_ = 70.9Multiple comparisons (of interest)Stimulation 1Neutral vs Paired1 *p* = 0.009; 95% CI: –93.5 to –10.6Neutral vs Paired2 *p* = 0.004; 95% CI: –98.6 to –15.7Paired 1 vs Paired2 *p* > 0.999; 95% CI: –36.4 to 46.5Stimulation 2Neutral vs Paired1 *p* < 0.001; 95% CI: –113 to –29.9Neutral vs Paired2 *p* < 0.001; 95% CI: –112 to –29Paired 1 vs Paired2 *p* > 0.999; 95% CI: –42.3 to 40.6CRNeutral vs Paired1 *p* = 0.998; 95% CI: –49.8 to 33.1Neutral vs Paired2 *p* > 0.999; 95% CI: –35.5 to 47.4Paired 1 vs Paired2 *p* = 0.938; 95% CI: –55.7 to 27.2
Extended Data Figure 8-1*Iiii*Time spent in paired1, paired2, and neutral compartments during the two NCP days for Vglut2-Cre/ChR2 mice	Normally distributed	RM one-way ANOVA followed by Tukey’s multiple comparison test	*N* = 5(M = 0, F = 5)	Compartment *p* = 0.018, *F*_(2,2)_ = 54.2Multiple comparisonsPaired1 vs Paired2 *p =* 0.951; 95% CI: –38.9 to 43.2Paired1 vs Neutral *p =* 0.023; 95% CI: –103 to –20.7Paired2 vs Neutral *p =* 0.021; 95% CI: –105 to –22.8

## Results

### NeuroD6 mRNA is found in a modest population of the medial VTA where it co-localizes extensively with dopaminergic markers and with a glutamatergic marker to minor degree

**Figure 1.**
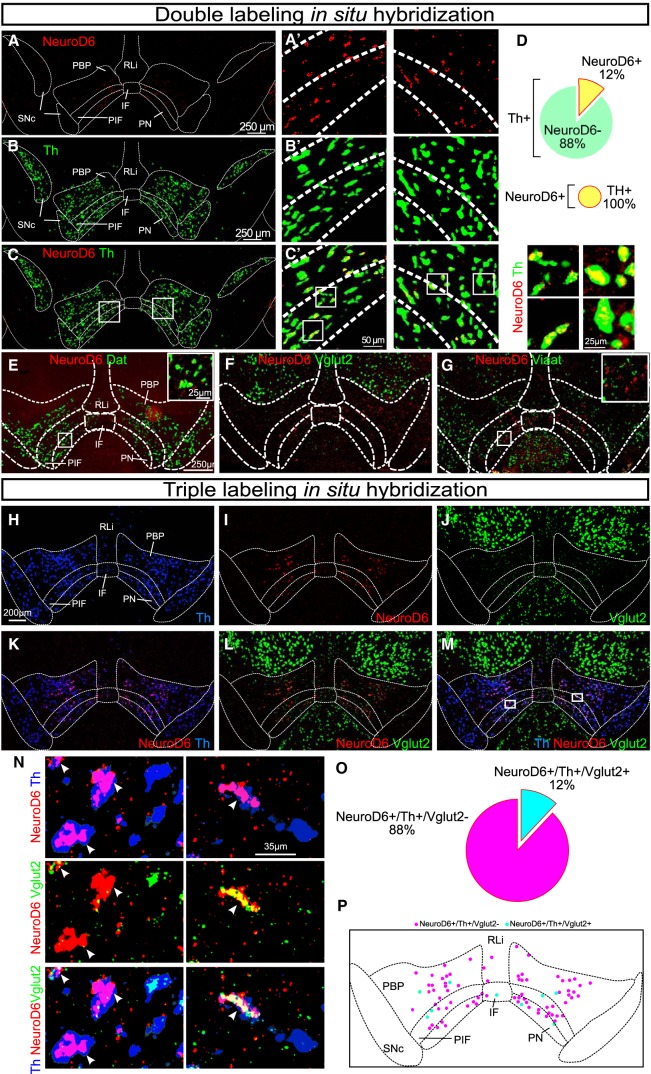
NeuroD6 mRNA is found in a modest population of the VTA, co-localizes with dopaminergic markers and partially with a glutamatergic marker. ***A–G***, Double FISH in the ventral midbrain of adult wild-type mice detecting the following mRNAs. ***A***, ***A’***, NeuroD6 (red). ***B***, ***B’***, Th (green). ***C***, ***C’***, NeuroD6 (red) and Th (green). Th/NeuroD6 mRNA overlap shown in yellow. Low magnification to the left; close-ups to the right. Schematic outline shows borders for SNc and subregions of VTA: PN, PIF, PBP, IF, RLi. ***D***, Quantification of percentage of NeuroD6-positive cells among all Th VTA cells; all NeuroD6 cells are positive for Th mRNA. ***E***, NeuroD6 (red) and Dat (green), inset with high magnification of Dat/NeuroD6 mRNA overlap (yellow). ***F***, NeuroD6 (red) and Vglut2 (green). ***G***, NeuroD6 (red) and Viaat (green), inset with high magnification of Viaat-negative/NeuroD6-positive (red). ***H–P***, Triple-labeling FISH in the ventral midbrain of adult wild-type mice detecting: (***H***) Th (blue); (***I***) NeuroD6 (red); (***J***) Vglut2 (green) mRNAs and their co-localization: (***K***) NeuroD6/Th; (***L***) NeuroD6/Vglut2; (***M***) Th/NeuroD6/Vglut2. Cellular closeups: (***N***) NeuroD6/Th (top), NeuroD6/Vglut2 (middle), Th/NeuroD6/Vglut2 (bottom). Arrows point to NeuroD6 mRNA-positive cells. ***O***, Quantification of percentage of NeuroD6+/Th+/Vglut2+ and NeuroD6+/Th+/Vglut2− neurons of the VTA. ***P***, Schematic illustration of distribution pattern of NeuroD6+/Th+/Vglut2+ and NeuroD6+/Th+/Vglut2− neurons within the VTA (same as shown with experimental data in ***M***). NeuroD6+/Th+/Vglut2- cells in magenta; NeuroD6+/Th+/Vglut2+ cells in cyan. VTA, ventral tegmental area; SNc, substantia nigra pars compacta; PBP, parabrachial pigmented nucleus; PN, paranigral nucleus; PIF, parainterfascicular nucleus; RLi, rostral linear nucleus; IF, interfascicular nucleus. FISH, fluorescent *in situ*; Dat, Dopamine transporter; Th, Tyrosine hydroxylase; Vglut2, Vesicular glutamate transporter 2; Viaat, Vesicular inhibitory amino acid transporter.

To address the distribution pattern and neurotransmitter identity of *NeuroD6*-expressing neurons, double-labeling FISH was first performed in which NeuroD6 mRNA (Fig. 1*A*,*C*) was compared to tyrosine hydroxylase (Th) mRNA encoding the rate-limiting enzyme (TH) of DA synthesis (Fig. 1*B*,*C*). Using the distribution pattern of Th mRNA as reference, DA neurons of the SNc and VTA were identified, including the paranigral (PN), parainterfascicular (PIF), parabrachial pigmented nucleus (PBP), interfascicular nucleus (IF), and rostral linear nucleus (RLi) subareas of the VTA (Fig. 1*A–C*). NeuroD6 mRNA was excluded from the SNc, but was detected in scattered VTA neurons. Most NeuroD6 neurons were found within the PN, PIF, and PBP subareas of the VTA, followed by fewer NeuroD6 neurons in the IF and RLi (Fig. 1*A*,*C*). Co-detection analysis showed that all neurons detected as positive for NeuroD6 mRNA within the PN, PIF, PBP, IF, and RLi were positive for Th mRNA (Fig. 1*C*). Quantification verified that 100% of NeuroD6 mRNA-positive cells in the PN/PIF, PBP, IF, and RLi were positive for Th mRNA, while 12% of all Th-expressing neurons within these VTA subareas contained NeuroD6 mRNA (Fig. 1*D*). To further address the dopaminergic identity of NeuroD6 neurons, co-detection of NeuroD6 mRNA with Dat mRNA, encoding the DA transporter (DAT), was performed. Similar to the overlap between NeuroD6 and Th, all neurons detected as positive for NeuroD6 mRNA in the VTA were positive for Dat mRNA (Fig. 1*E*). To further address the neurotransmitter identity of the NeuroD6-mRNA-positive VTA neurons, co-detection analyses of NeuroD6 mRNA with vesicular glutamate transporter 2 (Vglut2) and vesicular inhibitory amino acid transporter (Viaat) mRNAs were performed for identification of glutamatergic and GABAergic properties, respectively. NeuroD6 mRNA showed some co-localization with Vglut2 mRNA (Fig. 1*F*), while no or very few NeuroD6-positive cells in the VTA were detected as positive for Viaat mRNA (Fig. 1*G*). To address the overlap of NeuroD6 mRNA with Vglut2 and Th mRNA in detail, triple-labeling ISH of NeuroD6, Th and Vglut2 mRNAs was performed (Fig. 1*H–P*). This experiment confirmed that all NeuroD6 VTA neurons within the PN, PIF, PBP, IF, and RLi were detected as positive for Th (Fig. 1*H*,*K*,*N*) and that some NeuroD6 neurons co-localized with Vglut2 (Fig. 1*I*,*L*,*N*). Further, the experiment identified that these NeuroD6/Vglut2 double positive cells in the VTA were positive for Th mRNA (Fig. 1*J*,*M*,*N*). Quantification verified that 100% of NeuroD6 VTA neurons were positive for Th (NeuroD6+/Th+), and showed that 12% of these NeuroD6+/Th+ VTA neurons were also positive for Vglut2 mRNA. 12% thus displayed a NeuroD6+/Th+/Vglut2+ triple-positive molecular phenotype, while the remaining 88% of NeuroD6/Th neurons were negative for Vglut2 (NeuroD6+/Th+/Vglut2-; Fig. 1*O*). NeuroD6+/Th+/Vglut2+ and NeuroD6+/Th+/Vglut2- VTA neurons were distributed throughout the VTA with highest density in PN, PIF, and PBP subareas (Fig. 1*M*,*P*).

### Conditional ablation of the *Vmat2* gene in NeuroD6-Cre VTA neurons, a model for spatially restricted DA deficiency

**Figure 2.**
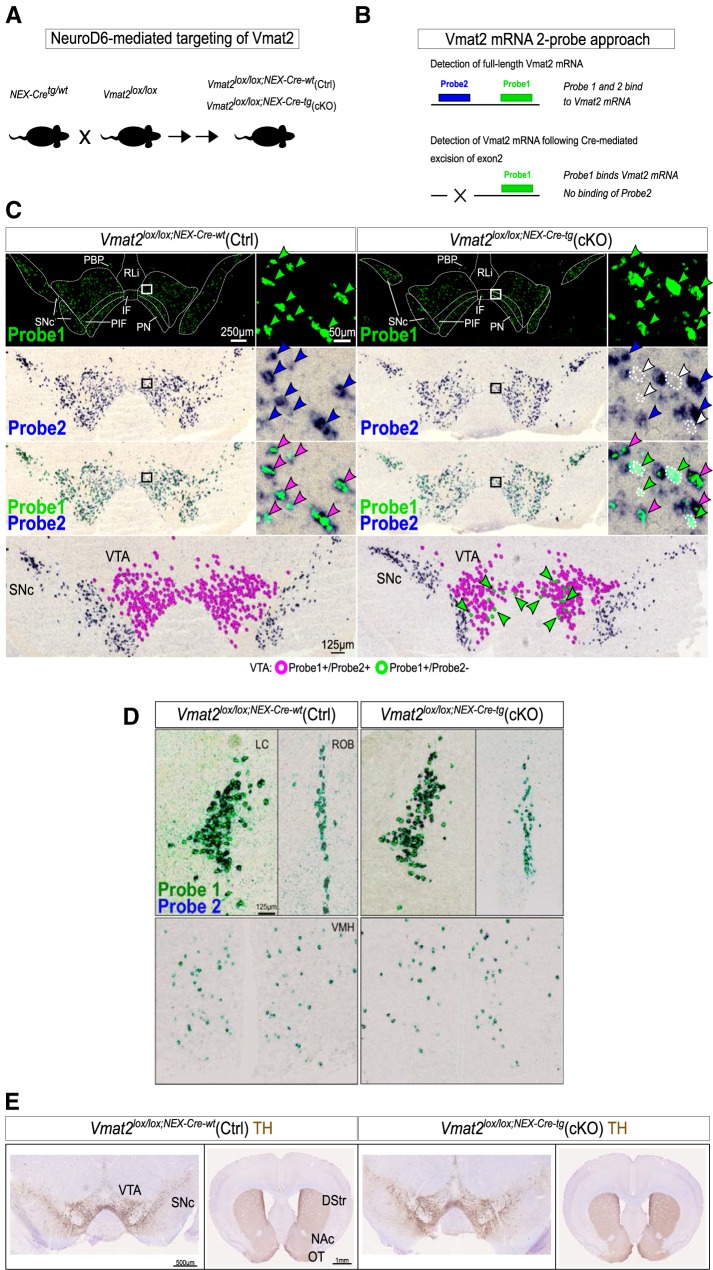
Conditional ablation of the Vmat2 gene in NEX-Cre neurons, a model for spatially restricted DA deficiency. ***A***, Breeding strategy for generation of mice gene-targeted for Vmat2 in VTA NEX-Cre neurons. NEX-Cre transgenic mice were mated to *Vmat2^lox/lox^* mice to generate NEX-Cre-positive mice homozygous for *Vmat2^lox/lox^* (*Vmat2^lox/lox;NEX-Cre-tg^*: cKO mice) and littermate control mice homozygous for *Vmat2^lox/lox^* and negative for the NEX-Cre transgene (*Vmat2^lox/lox;NEX-Cre-wt^*: Ctrl mice). ***B***, Two-probe approach for detection of Vmat2 mRNA by ISH. Probe 1 detects exons 6–15 and probe 2 detects exon 2 of the Vmat2 gene. Exon 2 is floxed in *Vmat2^lox/lox^* mice leading to failure of probe 2-binding to Vmat2 mRNA in cKO neurons. ***C***, Implementation of Vmat2 mRNA two-probe approach in *Vmat2^lox/lox;NEX-Cre-wt^* (Ctrl, left panel) and *Vmat2^lox/lox;NEX-Cre-tg^* (cKO, right panel) brains. Wild-type neurons are positive for both Vmat2 probes, while cKO neurons are only positive for probe 1 due to targeted deletion of exon 2 (detected by probe 2). Probe 1 detected in green and probe 2 detected in blue results in green-blue double-labeling in wild-type cells and green-only labeling in cKO cells. Green arrows point to green-only cells, i.e., VMAT2 cKO cells. ***D***, Vmat2 mRNA two-probe ISH in additional monoaminergic areas. ***E***, TH immunohistochemistry in Ctrl and cKO midbrain and striatum. LC, locus coeruleus; ROB, raphe nucleus obscurus; VMH, ventromedial hypothalamus; VTA, ventral tegmental area; SNc, substantia nigra pars compacta*;* DStr, dorsal striatum; NAc, nucleus accumbens; OT, olfactory tubercle. TH, Tyrosine hydroxylase; Vmat2/VMAT2, Vesicular monoamine transporter 2; Ctrl, control; cKO, conditional knockout.

To analyze the consequences of lost ability for vesicular packaging of DA in NeuroD6 VTA DA neurons, the *Slc18a2/Vmat2* gene encoding VMAT2 was targeted using a NeuroD6-Cre (NEX-Cre) transgenic mouse line. By breeding NEX-Cre mice with *Vmat2^lox/lox^* mice, *Vmat2^lox/lox;NEX-Cre-tg^* (cKO), and littermate control (Ctrl) mice were produced (Fig. 2*A*). Upon PCR-based analysis of genotype, brains from Ctrl and cKO mice were analyzed by ISH to verify loss of full-length Vmat2 mRNA in cKO mice. Due to the scarcity of NeuroD6-positive neurons in the VTA, a Vmat2 mRNA two-probe approach was used to allow detection of gene-targeted neurons. Vmat2 probe 1 was designed to detect all cells positive for Vmat2 mRNA, while Vmat2 probe 2 was designed to bind mRNA derived from exon 2, the exon targeted for recombination by Cre recombinase (Fig. 2*B*). In the ventral midbrain of control mice, probe 1 (green) and probe 2 (blue) were detected throughout the VTA and SNc areas with complete overlap (Fig. 2*C*, left panel). In the corresponding area of cKO mice, the majority of cells were positive for both probe 1 and probe 2 with complete overlap (Fig. 2*C*, right panel). However, throughout the PN, PIF, PBP, and IF VTA subareas, sparse cells showing green color only (probe 1) were detected, thus visualizing *Vmat2*-gene targeted cells among the mass of VTA DA neurons positive for both Vmat2 probes 1 and 2 (Fig. 2*C*, right panel). Having confirmed NEX-Cre-mediated recombination of the floxed *Vmat2* gene within scattered neurons of the VTA, other brain areas in which monoaminergic neurons reside were addressed by oligo ISH. Apart from the modest VTA population positive for NeuroD6 mRNA, NeuroD6 mRNA was not detected within any other monoaminergic cell group, identified by Th and Vmat2 mRNA (Extended Data Fig. 2-1). However, as previously reported (Goebbels et al., 2006), NeuroD6 was abundant in several non-dopaminergic brain structures, primarily the cerebral cortex and hippocampus (Extended Data Fig. 2-1). In accordance with the lack of NeuroD6 in all monoaminergic cell groups apart from the VTA, Vmat2 probe 1 and probe 2 showed complete overlap in these areas, including locus coeruleus, ventromedial hypothalamus, and nucleus raphe obscurus, while none displayed labeling from probe 1 only (Fig. 2*D*). These experiments showed that in cKO mice, Vmat2 mRNA was selectively ablated within the VTA. To address whether the targeted deletion of *Vmat2* in NeuroD6 neurons of the VTA affected the morphology of the midbrain DA system, distribution patterns of Th mRNA and TH protein were addressed, none of which revealed any gross anatomic difference in the dopaminergic system between Ctrl and cKO mice (Fig. 2*E*; Extended Data Fig. 2-1).

10.1523/ENEURO.0066-19.2019.f2-1 Extended Data Figure 2-1 ISH for detection of Th, NeuroD6, and Vmat2 mRNA. Analysis of Th, NeuroD6, and Vmat2 (two probes, covering mRNA derived from exon 1 and exon 2, respectively) mRNAs using radioactively labeled oligo-probes on sections throughout the whole brains of *Vmat2^lox/lox/NEX-Cre-wt^* (Ctrl) and *Vmat2^lox/lox/NEX-Cre-tg^* (cKO) mice. Download Figure 2-1, EPS file.

### Heightened locomotor response to psychostimulants upon gene-targeting of *Vmat2* in NEX-Cre VTA neurons

**Figure 3.**
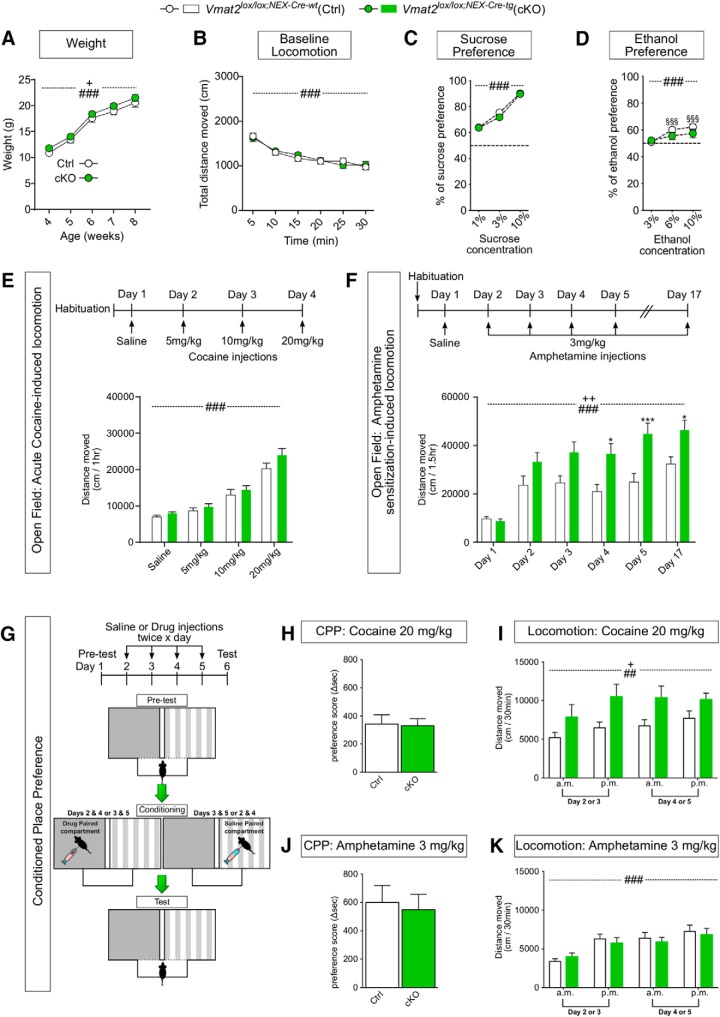
Altered responsiveness to psychostimulants upon ablation of Vmat2 gene expression in NeuroD6 VTA neurons. Color coding: *Vmat2^lox/lox;NEX-Cre-wt^* (Ctrl) in white; *Vmat2^lox/lox;NEX-Cre-tg^*(cKO) in green. ***A***, Weight curve for Ctrl (*N* = 14) and cKO (*N* = 23) mice. Data presented as mean weight in grams for each week ± SEM (**+***p* < 0.05 effect of genotype; ###*p* < 0.001, effect of age). ***B***, Baseline locomotion in novel environment. Ctrl (*N* = 17) and cKO (*N* = 17). Data expressed as mean distance moved in 5-min bins ± SEM (###*p* < 0.001, effect of time). ***C***, Sucrose preference expressed as percentage of preference for sucrose over tap water ± SEM. Ctrl (*N* = 14) and cKO (*N* = 21; ###*p* < 0.001 effect of sucrose concentration). ***D***, Ethanol preference expressed as percentage of preference for ethanol solution over tap water ± SEM. Ctrl (*N* = 14) and cKO (*N* = 14; ###*p* < 0.001 effect of ethanol concentration, §§§*p* < 0.001 3% vs 6% and 10% in ctrl mice). ***E***, Cocaine-induced locomotion. Top, Administration schedule. Bottom, Average distance moved 1 h after injection of saline and 5, 10, 20 mg/kg of cocaine; Ctrl (*N* = 14) and cKO (*N* = 21) mice. Data expressed as total distance moved during the 1-h recording period ± SEM (###*p* < 0.001 effect of session). ***F***, Amphetamine-induced locomotion. Top, Administration schedule. Bottom, Average distance moved 1.5 h after injection; Ctrl (*N* = 17) and cKO mice (*N* = 17). Data presented as mean of total distance moved in cm ± SEM for each session; **++***p* < 0.01 effect of genotype, ###*p* < 0.001 effect of session, *p < 0.05 and ***p < 0.001 cKO versus Ctrl. ***G***, CPP. Illustration of setup and administration schedule. ***H***, ***J***, Preference score displayed as Δsec, the difference between time spent in drug-paired compared during pretest and test ± SEM, positive value indicates preference (cocaine: Ctrl *N* = 12, cKO *N* = 15; amphetamine: Ctrl *N* = 13, cKO *N* = 16). ***I***, ***K***, Cocaine-induced and amphetamine-induced locomotion during conditioning in the CPP setup displayed as distance moved in 30 min ± SEM (cocaine: Ctrl *N* = 12, cKO *N* = 15; amphetamine: Ctrl *N* = 15, cKO *N* = 17, **+***p* = 0.031 effect of genotype, ##*p* = 0.006, ###*p* < 0.001 effect of session). Ctrl, Control; cKO, conditional knockout; CPP, Conditioned place preference.

To address whether it is possible to dissociate an explicit behavioral role of DA neurotransmission exerted by NeuroD6 VTA DA neurons from the range of behaviors ascribed to the mDA system, *Vmat2^lox/lox;NEX-Cre-tg^* cKO mice were tested in a battery o*f* tests relevant to the mDA system and compared to *Vmat2^lox/lox;NEX-Cre-wt^* Ctrl mice. To assess body weight, mice were weighed every week from weaning to adulthood. cKO mice were similar to their Ctrl littermates weight-wise (effect of age: *F*_(4158)_ = 79.8, *p* < 0.001; genotype: *F*_(1158)_ = 4.67 *p* = 0.032; no age × genotype interaction, no *post hoc* differences between genotypes; Fig. 3*A*).

#### Baseline locomotion

The habituation response to a novel environment, a gross measure of stress and exploratory behavior, was addressed. Both Ctrl and cKO mice showed the same rate of reaching a stable plateau in baseline locomotion (effect of time: *F*_(5160)_ = 69.5, *p* < 0.001; effect of genotype: *F*_(1,32)_ = 0.00912, *p* = 0.535; Fig. 3*B*).

#### Sucrose and ethanol preference

A sucrose bottle preference test was next performed. Both Ctrl and cKO mice preferred the ascending concentrations of sucrose solutions over water (effect of concentration: *F*_(2,66)_ = 151, *p* < 0.001), but no differences between the genotypes were observed (effect of genotype: *F*_(1,33)_ = 1.12, *p* = 0.297; Fig. 3*C*). The rewarding effect of alcohol was subsequently measured by using increasing concentrations of ethanol (3%, 6%, 10%) presented in a bottle preference test. Again, both Ctrl and cKO mice preferred the presented reward over water (effect of concentration: *F*_(2,52)_ = 14.2, *p* < 0.001), but there was no difference between the genotypes (effect of genotype: *F*_(1,26)_ = 0.969, *p* = 0.334). However, *post hoc* analysis showed that Ctrl mice significantly preferred the 6% and 10% concentrations over the 3% solution (§§§*p* < 0.001 3% vs 6% and 10% ethanol in ctrl mice), while a trend toward significant differences in cKO mice was observed only between the 3% and 10% ethanol solutions (3% vs 10%: *p* < 0.072; Fig. 3*D*).

#### Cocaine-induced and amphetamine-induced locomotion

To address locomotor responses on psychostimulant-injections, cocaine and amphetamine administration protocols were applied and locomotion was measured. Following administration of acute ascending doses of cocaine (5, 10, and 20 mg/kg), both Ctrl and cKO mice displayed increased locomotion in a dose-dependent manner; however, no significant differences were observed between genotypes (effect of session: *F*_(3,99)_ = 108, *p* < 0.001; genotype, *F*_(1,33)_ = 1.65, *p* = 0.208; session × genotype interaction: *F*_(3,99)_ = 1, *p* = 0.396; Fig. 3*E*). Next, an amphetamine sensitization protocol was applied. All mice responded to amphetamine with hyperlocomotion, but the effect was significantly higher in cKO mice than control mice in days 4, 5, and 17 of the experiment (effect of day: *F*_(5160)_ = 40.9, *p* < 0.001; genotype, *F*_(1,32)_ = 9.09, *p* = 0.005; day × genotype interaction: *F*_(5160)_ = 4.79; *p* < 0.001; ctrl vs cKO day 4 *p* = 0.011, day 5 *p* < 0.001, day 17 *p* = 0.029; Fig. 3*F*).

#### CPP

To study the reinforcing effects of psychostimulants, a CPP procedure was applied (Fig. 3*G*). Both Ctrl and cKO mice showed preference for the cocaine-paired or amphetamine-paired compartment over the saline-paired compartment with no significant difference between genotypes (ctrl vs cKO cocaine: *p* = 0.860, amphetamine *p* = 0.744; Fig. 3*H*,*J*). In addition to preference, locomotion was monitored during the conditioning sessions. cKO mice displayed increased locomotor responses after repeated administration of cocaine compared to Ctrl mice (effect of session; *F*_(3,75)_ = 4.4, *p* = 0.006; effect of genotype *F*_(1,25)_ = 5.2, *p* = 0.031, no differences in *post hoc* analysis; Fig. 3*I*). In contrast, in the CPP paradigm, repeated administration of amphetamine did not induce elevated locomotion in cKO over Ctrl mice (effect of session; *F*_(3,85)_ = 24.0, *p* < 0.001; effect of genotype *F*_(1,30)_ = 0.0631, *p* = 0.803; Fig. 3*K*).

### NeuroD6 mRNA co-localizes partly with Calb2 mRNA, but Calb2 mRNA is abundant throughout VTA and SNc

**Figure 4:**
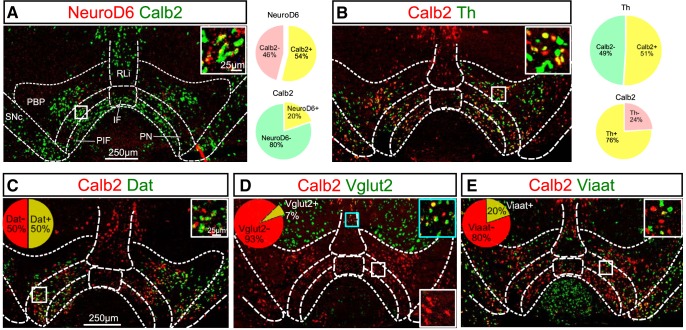
NeuroD6 mRNA co-localizes partly with Calb2 mRNA, but Calb2 mRNA is abundant throughout the VTA and SNc. Double-labeling FISH in the ventral midbrain of adult wild-type mice detecting the following mRNAs. ***A***, NeuroD6 (red) and Calb2 (green), inset with high magnification of overlap (yellow), pie charts illustrating quantification of overlap between NeuroD6 and Calb2. ***B***, Calb (red) and Th (green), inset with high magnification of overlap (yellow), pie charts illustrating quantification of overlap between Th and Calb2. ***C***, Calb2 (red) and Dat (green), inset with high magnification of Dat/Calb2 mRNA overlap (yellow), pie chart illustrating quantification of overlap between Th and Calb2. ***D***, Calb2 (red) and Vglut2 (green), inset with high magnification of Vglut2/Calb2 mRNA overlap (yellow) in blue square and Vglut2-negative/Calb2-positive (red) in white square, pie chart illustrating quantification of overlap between Th and Calb2. ***E***, Calb2 (red) and Viaat (green), inset with high magnification of Viaat negative or positive (red, yellow, green) in white square. VTA, ventral tegmental area; SNc, substantia nigra pars compacta; PBP, parabrachial pigmented nucleus; PN, paranigral nucleus; PIF, parainterfascicular nucleus; RLi, rostral linear nucleus; IF, interfascicular nucleus. Calb2, Calbindin 2 (Calretinin); Dat, Dopamine transporter; Th, Tyrosine hydroxylase; Vglut2, Vesicular glutamate transporter 2; Viaat, Vesicular inhibitory amino acid transporter; FISH, fluorescent *in situ* hybridization.

To further characterize the molecular identity of NeuroD6 VTA neurons, FISH was next used to address the putative overlap between NeuroD6 and Calb2 mRNAs. Distribution patterns of NeuroD6 and Calb2 mRNAs within midbrain DA neurons were recently described without addressing their putative overlap (Viereckel et al., 2016). In contrast to the selective localization of NeuroD6 mRNA within the VTA and its exclusion from the SNc, Calb2 mRNA was abundant in both VTA and SNc (Fig. 4*A*). The restricted number of NeuroD6 neurons in the VTA showed partial overlap with Calb2 mRNA: 54% of all NeuroD6 VTA neurons were positive for Calb2 mRNA while 20% of Calb2 neurons expressed NeuroD6 mRNA (Fig. 4*A*). Further quantification within the VTA showed that Calb2 mRNA was detected in 51% of all Th-neurons, with a similar match of Calb2/Dat co-localization at 50% (Fig. 4*B*,*C*). Some Calb2 neurons in the VTA were positive for Vglut2 mRNA (7%; Fig. 4*D*), while 20% of all Calb2 neurons in the VTA were positive for Viaat mRNA (Fig. 4*E*).

### Spatially restricted striatal innervation by NeuroD6-Cre and Calb2-Cre VTA neurons

**Figure 5.**
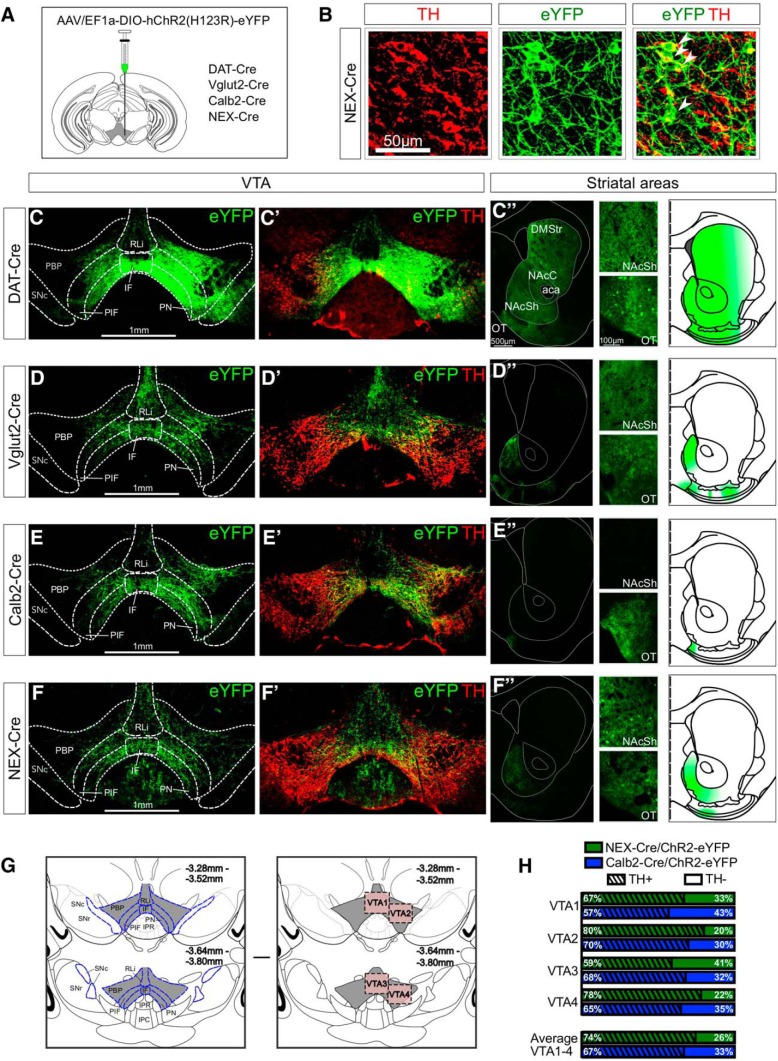
Spatially restricted striatal innervation by NeuroD6 and Calb2 VTA neurons. ***A***, Schematic illustration of stereotaxic injection into VTA of Cre-dependent *DIO-ChR2-eYFP* DNA construct packaged into AAV. ***B***, Representative VTA neurons immunopositive for TH (red), YFP (green), or both (yellow; *DIO-ChR2-eYFP*-injected NEX-Cre mice). ***C–F***, Representative pictures of VTA (left panels) and striatal complex (right panels) in *DIO-ChR2-eYFP*-injected DAT-Cre (***C–C’’***), Vglut2-Cre (***D–D’’***), Calb2-Cre (***E–E’’***), and NEX-Cre (***F–F’’***) mice. Panel far right, Schematic summary of striatal innervation pattern. Additional target areas listed in Table 2. Quantification of YFP and TH immunofluorescent overlap: schematic illustration of four representative VTA areas selected for counting, shown as squares and labeled VTA 1–4 (***G***). Results of quantifications shown in histograms for each VTA area and the total sum (***H***). PBP, parabrachial pigmented nucleus; PN, paranigral nucleus; PIF, parainterfascicular nucleus; RLi, rostral linear nucleus; IF, interfascicular nucleus; SNc, substantia nigra pars compacta; SNr, substantia nigra pars reticulata; IPR, interpeduncular nucleus, rostral subnucleus; IPC, interpeduncular nucleus, caudal subnucleus; DMStr, dorsomedial striatum; NAcC, nucleus accumbens core; NAcSh, nucleus accumbens shell; aca; anterior commissure, anterior part; OT, olfactory tubercle. DAT, Dopamine transporter, Calb2, Calbindin 2 (Calretinin); NEX, NeuroD6; Vglut2; Vesicular glutamate transporter 2; Th, Tyrosine hydroxylase; ChR2; Channelrhodopsin 2; eYFP, enhanced Yellow fluorescent protein.

Next, to allow analysis of projections, signaling properties and behavioral regulation of NEX-Cre and Calb2-Cre VTA neurons, optogenetics was implemented. Upon infusion of viral particles carrying a double-floxed *DIO-ChR2-eYFP* genetic construct encoding both Channelrhodopsin (ChR2) and the enhanced yellow fluorescent protein (eYFP) into the VTA, mice were analyzed in different parameters. DAT-Cre and Vglut2-Cre transgenic mice were used as controls based on their representation of VTA and SNc dopaminergic and glutamatergic neurons, respectively (Stuber et al., 2010; Hnasko et al., 2012; Pascoli et al., 2015; Qi et al., 2016; Yoo et al., 2016). First, Cre-driven expression of the *DIO-ChR2-eYFP* construct in DAT-Cre, Vglut2-Cre, Calb2-Cre and NEX-Cre mice was analyzed histologically by comparing YFP with TH immunolabeling (Fig. 5*A*). In DAT-Cre, Vglut2-Cre, Calb2-Cre, and NEX-Cre mice, YFP fluorescent labeling was identified in the VTA, verifying the activity of each Cre-driver to recombine the floxed optogenetic construct (Fig. 5*B–F*). YFP co-localized extensively with TH in the VTA. YFP was strongest and most abundant in the VTA of DAT-Cre mice, while Vglut2-Cre, Calb2-Cre, and NEX-Cre mice all showed lower amount of cells positive for YFP (Fig. 5*B–F*). Next, to reveal target areas, sections throughout the entire brain of all four Cre-driver mouse lines were analyzed and compared. Some target areas were the same for all four Cre-drivers, including the NAcSh and ventral pallidum, while others differed, such as the distribution within the medial and lateral habenula (Table 2). Overall, the density of YFP-positive fibers was substantially lower in NEX-Cre and Calb2-Cre mice than in DAT-Cre and Vglut2-Cre mice. Following analysis of sections throughout the brain, the VTA and striatum were analyzed in more detail. DAT-Cre mice showed strong cellular YFP labeling within all VTA subareas (sparse in RLi) and within the SNc, primarily on the injected side (Fig. 5*C–C’’*). YFP-positive fibers were distributed across the striatal complex including primarily the dorsomedial striatum, NAcSh, NAc core and the olfactory tubercle (OT; Fig. 5*C–C’’*). Vglut2-Cre mice showed YFP-labeled cell bodies primarily in the medial VTA with fibers innervating the NAc and OT (Fig. 5*D–D’’*). Next, Calb2-Cre and NEX-Cre mice were addressed. Calb2-Cre mice showed similar distribution of YFP-labeling as DAT-Cre within VTA, but the density was sparser than in DAT-Cre mice (Fig. 5*E–E’’*). YFP-positive fibers in the striatal complex were detected in the OT (Fig. 5*E–E’’*). NEX-Cre mice showed a low number of YFP cells in the VTA (Fig. 5*F–F’’*), in accordance with the modest distribution of endogenous NeuroD6 mRNA described above. Weak YFP fluorescence was detected in fibers throughout the NAcSh and OT (Fig. 5*F–F’’*). The distribution pattern of YFP-positive cells in the VTA of NEX-Cre mice was similar as the distribution of endogenous NeuroD6 mRNA. However, the YFP appeared more abundant than the above analyzed NeuroD6 mRNA. Quantification was performed to address the overlap between YFP and TH. The majority of NEX-Cre/YFP and Calb2-Cre/YFP neurons showed TH immunoreactivity; however, for both Cre-lines, a number of YFP cells were negative for TH (NEX-Cre/ChR2: TH+: 4013 ± 21.72, eYFP+ 965 ± 4.17, double: 715 ± 3.24; Calb2-Cre/ChR2: TH+: 4187 ± 18.9, eYFP+: 1396 ± 6.04, double: 939 ± 4.69). In total, 74% of NEX-Cre and 67% of Calb2-Cre neurons showed overlap between YFP and TH (Fig. 5*G*,*H*).

**Table 2.** Projection areas of VTA neurons represented in NEX-Cre and Calb2-Cre mice compared with DAT-Cre and Vglut2-Cre mice

Area	Cre-driver			
	DAT	Vglut2	Calb2	NEX
Anterior olfactory area	+	+	+	+
Medial prefrontal cortex (infralimbic, prelimbic, and anterior cingulate cortices)	+	+	+	+
(Medial) orbital cortex	+	+	+	+
Nucleus accumbens shell	+	+	(+)	+
Nucleus accumbens core	+	-	-	+
Dorsomedial Striatum	+	-	-	-
Olfactory tubercle	+	+	+	+
Cingulate cortex	+	+	+	+
Septum/septal nuclei	+	+	-	+
Diagonal band of Broca	+	+	+	+
Ventral pallidum	+	+	+	+
Bed nuclei of the stria terminalis	+	+	+	+
Preoptic area	+	+	+	+
Lateral habenula	+	+	-	-
Medial habenula	-	-	+	+
Hippocampus	-	-	-	+
Dentate gyrus	-	-	-	+
Amygdala	+	+	+	+
Hypothalamic area	+	+	-	+

Summary of projection areas for VTA neurons virally injected with optogenetic constructs (*DIO-ChR2-eYFP*) in DAT-Cre, Vglut2-Cre, Calb2-Cre, and NEX-Cre mice, respectively, and detected as YFP-positive fibers. + indicates presence of YFP-positive fibers; - indicates absence of YFP-positive fibers; (+) indicates low presence of fibers.

### Optogenetic stimulation in striatal target areas of NeuroD6 and Calb2 VTA neurons verifies DA release

**Figure 6:**
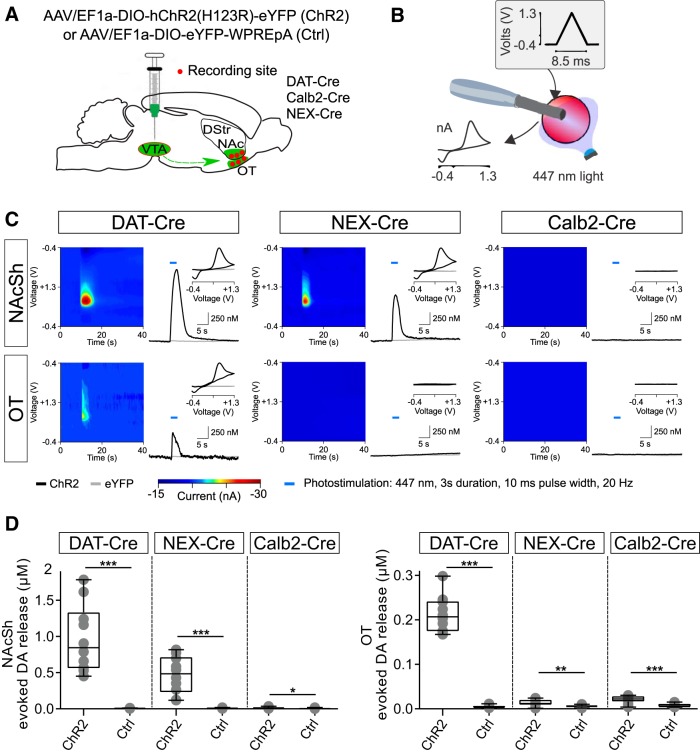
Optogenetic stimulation in striatal target areas of NeuroD6 and Calb2 VTA neurons verifies DA release. ***A***, Schematic representation of stereotaxic injection into VTA of Cre-dependent *DIO-ChR2-eYFP* and *DIO-eYFP* (Ctrl); FSCV recording sites within NAcSh and OT (red dots). ***B***, Illustration of the experimental setup. ***C***, Representative light-evoked DA recordings from injected DAT-Cre (left), NEX-Cre (middle), and Calb2-Cre (right) mice in the NAcSh (top) and the OT (bottom). ***D***, Quantification of photostimulation-evoked DA release in the NAc shell (left) and OT (right); *N* = 10 recording sites per group for each region. Mice used for the recordings: DAT-Cre/ChR2 *N* = 2, DAT-Cre/eYFP *N* = 2, NEX-Cre/ChR2 *N* = 3, NEX-Cre/eYFP *N* = 2 Calb2-Cre/ChR2 *N* = 3, and Calb2-Cre/eYFP *N* = 2. Box and whisker plots, Center lines indicate medians, box edges represent the interquartile range, whiskers extend to the minimal and maximal values (*p < 0.05, **p < 0.01, ***p < 0.001 ChR2 vs ctrl). DStr, dorsal striatum; NAcSh, nucleus accumbens shell; OT, olfactory tubercle. DAT, Dopamine transporter; Calb2, Calbindin 2 (Calretinin); ChR2; Channelrhodopsin 2; eYFP, enhanced Yellow fluorescent protein; NEX, NeuroD6.

To address neurotransmitter release, extracellular DA concentration upon optogenetic stimulation was recorded using FSCV in slice preparations. DAT-Cre, NEX-Cre, and Calb2-Cre mice injected with the same *DIO-ChR2-eYFP* construct as above (Fig. 6*A*) were analyzed upon photostimulation and subsequent recording within the NAcSh and OT (Fig. 6*B*). Cre-mice injected with *DIO-eYFP* were used as controls. DA levels (∼1 μM) were readily recorded upon photostimulation in both the NAcSh of DIO-ChR2 injected DAT-Cre (0.9699 ± 0.1471 μM) and NEX-Cre mice (0.4701 ± 0.08043 μM), while a lower signal was obtained in the NAcSh of Calb2-Cre/ChR2 mice (0.01509 ± 0.002845 μM; Fig. 6*C*,*D*). Upon photostimulation and recording in the OT, lower DA levels (∼200 nM) than those measured in the NAcSh were obtained in DAT-Cre/ChR2 mice (0.2129 ± 0.01291 μM) while even smaller levels were detected in both Calb2-Cre/ChR2 (0.02097 ± 0.002712 μM) and NEX-Cre/ChR2 mice (0.01362 ± 0.002304 μM; Fig. 6*C*,*D*). Despite comparably low in size, all DA levels recorded in mice expressing the ChR2-YFP were significantly larger than in mice injected with the control virus (DAT-Cre, NAcSh ChR2: 0.9699 ± 0.1471 μM, eYFP: 0.006802 ± 0.0008813 μM, *t*_(9)_ = 6.55 *p* < 0.0001, OT ChR2 0.2129 ± 0.01291 μM vs eYFP 0.004649 ± 0.0009871 μM, *t*_(9)_ = 16.08 *p* < 0.0001; NEX-Cre, NAcSh ChR2: 0.4701 ± 0.08043 μM, eYFP: 0.0102 ± 0.001682 μM, *t*_(9)_ = 5.716 *p* < 0.0001, OT ChR2: 0.01362 ± 0.002304 μM, eYFP: 0.005791 ± 0.0008003 μM, *t*_(9)_ = 3.209 *p* = 0.0049; Calb2-Cre, NacSh ChR2: 0.01509 ± 0.002845 μM, eYFP: 0.006087 ± 0.001746 μM, *t*_(9)_ = 2.696 *p* = 0.0148, OT ChR2: 0.02097 ± 0.002712 μM, eYFP 0.007081 ± 0.001315 μM, *t*_(9)_ = 4.607 *p* = 0.0002; Fig. 6*C*,*D*).

### Optogenetic stimulation in striatal target areas of NeuroD6 and Calb2 VTA neurons reveals a glutamatergic postsynaptic response

**Figure 7.**
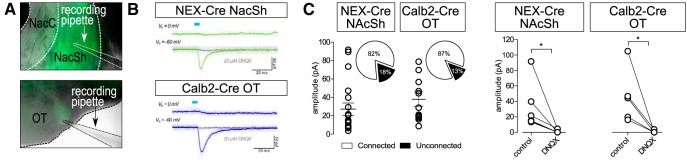
Optogenetic stimulation in striatal target areas of NeuroD6 and Calb2 VTA neurons reveals glutamatergic postsynaptic responses. ***A***, Representative picture from patch-clamp slice electrophysiology in NAcSh of NEX-Cre mice and OT of Calb2-Cre mice injected with *DIO-ChR2-eYFP*. ***B***, Representative traces of photostimulation-evoked postsynaptic currents recorded from NAcSh cells from NEX-Cre and OT cells from Calb2-Cre mice injected with *DIO-ChR2-eYFP*. ***C***, Pie charts represent the percentage of cells showing EPSCs (white) versus negative (black) upon photostimulation of terminals in the NAcSh (*N* = 18 cells from four mice) of NEX-Cre mice, and OT (*N* = 14 cells from 4 mice) of Calb2-Cre mice. The *y*-axis shows amplitude in pA; each circle represents one cell, and bold lines the mean amplitude ± SEM. ***D***, Patch-clamp recordings pre-bath (control) and post-bath application of DNQX upon photostimulation in NAcSh of NEX-Cre/ChR2 (left, *N* = 6 cells from three mice) and OT of Calb2-Cre/ChR2 mice (right, *N* = 5 cells from three mice). Each circle represents one cell (*p < 0.05 control vs DNQX). NAcSh, nucleus accumbens shell; OT, olfactory tubercle. Calb2, Calbindin 2 (Calretinin); NEX; NeuroD6; ChR2; Channelrhodopsin 2; eYFP, enhanced Yellow fluorescent protein.

To address the presence of postsynaptic currents in NAcSh and OT neurons upon optogenetic activation, patch clamp electrophysiology was implemented in NEX-Cre and Calb2-Cre injected with *DIO-ChR2-eYFP* (Fig. 7). Upon optogenetic stimulation, 82% of neurons in the NAcSh NEX-Cre mice (18 out of 22 cells) and 87% of OT neurons in Calb2-Cre mice (13 out of 15 cells) showed EPSCs (NEX-Cre NAcSh, mean amplitude 28 ± 6.8 pA; Calb2-Cre OT, mean amplitude 39 ± 7.7 pA; Fig. 7*B*,*C*). In both cases, EPSCs were almost completely abolished after bath application of 10 μM the AMPA receptor antagonist DNQX, demonstrating that the recorded currents are AMPA receptor-mediated (NEX-Cre NAcSh mean amplitude, control: 33 ± 13 pA, DNQX 1.5 ± 0.96 pA *t*_(5)_ = 2.602 *p* = 0.0481; Calb2-Cre OT, mean amplitude, control: 46 ± 16 pA, DNQX: 0.74 ± 0.74 pA *t*_(4)_ = 2.867 *p* = 0.0456; Fig. 7*D*). The synaptic delay of the EPSCs was short (NEX-Cre NAcSh 3.3 ± 0.25 ms; Calb2-Cre OT 3.6 ± 0.21 ms). In contrast, the mean decay time was longer in the OT than in NAcSh (NEX-Cre NAcSh 5.3 ± 0.5 ms; Calb2-Cre OT 7.8 ± 0.62 ms). No inhibitory/GABA-receptor-mediated currents were observed during recordings in either NEX-Cre or Calb2-Cre mice (Fig. 7*B*).

### Optogenetic activation of NeuroD6 VTA neurons, but not Calb2 VTA neurons, induces place preference

**Figure 8.**
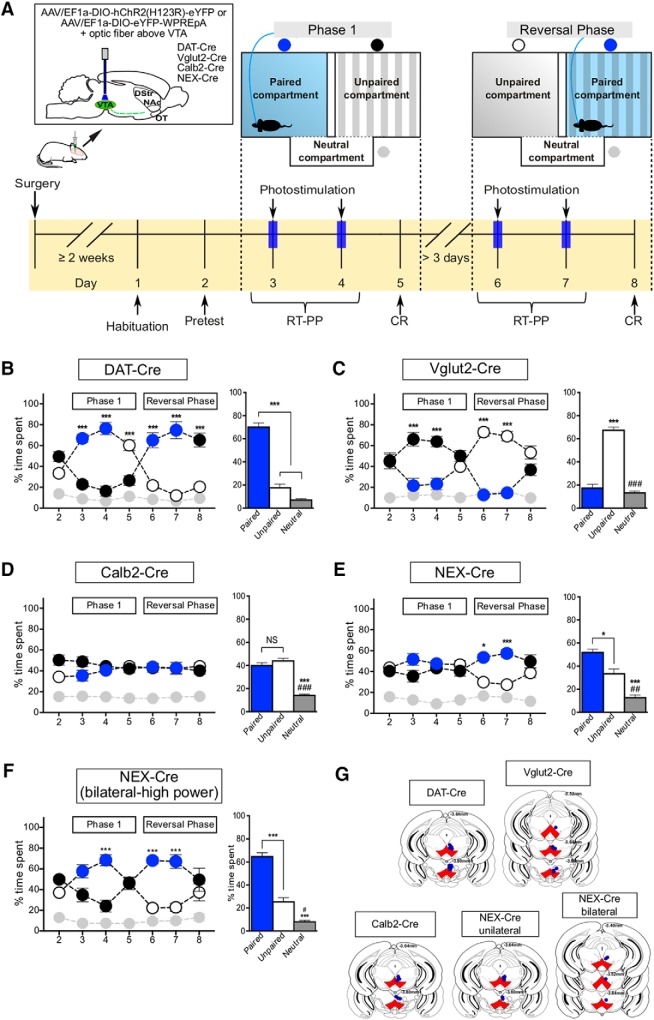
Optogenetic activation of NeuroD6 VTA neurons, but not Calb2 VTA neurons, induces place preference. ***A***, Schematic drawing of stereotaxic injection into VTA of Cre-dependent *DIO-ChR2-eYFP* and of experimental setup for RT-PP analysis. ***B–F***, Time spent in light-paired (blue), unpaired (white during phase 1, black during reversal phase), and neutral (gray) compartments shown as mean percentage of time spent in each compartment ± SEM (left; *p < 0.05, ***p < 0.001 paired vs unpaired compartment); average percentage of time spent in each compartment during days 3, 4, 6, and 7 ± SEM (bar graphs; right; *p < 0.05, ***p < 0.001 vs light-paired compartment; #*p* < 0.05, ##*p* < 0.01, ###*p* < 0.001 vs unpaired compartment). DAT-Cre *N* = 10; Vglut2-Cre *N* = 7; Calb2-Cre *N* = 7; NEX-Cre *N* = 5. ***F***, High-power stimulation of bilaterally injected NEX-Cre mice (*N* = 4). ***G***, Schematic illustration of optical fiber placement in mice analyzed in RT-PP analysis. NS, non-significant. DAT, Dopamine transporter; Calb2, Calbindin 2 (Calretinin); NEX, NeuroD6; Vglut2; Vesicular glutamate transporter 2; ChR2; Channelrhodopsin 2; eYFP, enhanced Yellow fluorescent protein.

Finally, *in vivo* optogenetic stimulation in the VTA of NEX-Cre and Calb2-Cre mice was applied to assess whether this would induce place preference behavior. Again, DAT-Cre and Vglut2-Cre mice were used as references for comparison to Calb2-Cre and NEX-Cre mice. Mice received *DIO-ChR2-eYFP* or *DIO-eYFP* (control) injection and implantation of optic fibers above the VTA (Fig. 8*A*,*G*), and were analyzed for RT-PP and CR (Fig. 8*A*).

#### Analysis of RT-PP and CR in DAT-Cre and Vglut2-Cre mice

DAT-Cre mice displayed a significant preference to the light-paired compartment on every day of stimulation (effect of compartment *F*_(2,18)_ = 51.8, *p* < 0.001; day × compartment interaction *F*_(12,108)_ = 33, *p* < 0.001, *** *p* < 0.001 paired vs unpaired compartment; Fig. 8*B*, left). This place preference was also evident when the effect of stimulation was averaged for the four experimental days (effect of compartment *F*_(2,6)_ = 166, *p* < 0.001, ***p < 0.001 vs paired compartment; Fig. 8*B*, right). In the absence of stimulation, on days 5 and 8, DAT-Cre mice demonstrated a CR for the previous light-paired compartment (***p < 0.001 paired vs unpaired; Fig. 8*B*). Control mice (DAT-Cre negative or DAT-Cre injected with *DIO-eYFP*) did not display any preference toward the stimulation [effect of compartment *F*_(2,4)_ = 4.26, *p* = 0.102; day × compartment interaction, *F*_(12,24)_ = 0.898 *p* = 0.562, *p < 0.05 paired vs unpaired compartment (Extended Data Fig. 8*-*1*A*, left); effect of compartment *F*_(2,6)_ = 48.7 *p* < 0.001, ***p < 0.001 ###*p* < 0.001 neutral versus paired and unpaired, respectively (Extended Data Fig. 8*-*1*A*, right); effect of compartment *F*_(2,4)_ = 27.9, *p* = 0.004, day × compartment interaction *F*_(12,24)_ = 0.767 *p* = 0.677, ***p < 0.001 paired vs unpaired compartment; right: effect of compartment *F*_(2,6)_ = 2.97, *p* = 0.127 (Extended Data Fig. 8-1*B*, left); effect of compartment *F*_(2,10)_ = 18.6, *p* < 0.001, day × compartment interaction *F*_(12,60)_ = 0.963, *p* = 0.494 (Extended Data Fig. 8-1*C*, left); effect of compartment *F*_(2,6)_ = 9.27, *p* = 0.015, *p < 0.05 #*p* < 0.05 neutral vs paired and unpaired, respectively (Extended Data Fig. 8-1*C*, right)]. These results were in accordance with the literature (Yoo et al., 2016) and thereby validated the experimental setup. In contrast to the strong place preference induced by stimulation in DAT-Cre mice, Vglut2-Cre mice analyzed in the same setup displayed a preference for the unpaired compartment [effect of compartment *F*_(2,12)_ = 40.9, *p* < 0.001 and day × compartment interaction *F*_(12,72)_ = 16.1, *p* < 0.001, ***p < 0.001 paired vs unpaired (Fig. 8*C*, left); effect of compartment *F*_(2,6)_ = 162, *p* < 0.001, ***p < 0.001 versus paired, ###*p* < 0.001 vs unpaired (Fig. 8*C*, right)]. To further verify this observation, the protocol was modified so that the mice would receive photostimulation upon entry to either one of the main compartments but not upon entry into the interconnecting neutral compartment (NCP; Extended Data Fig. 8-1*Ii*). Once again, Vglut2-Cre mice preferred to spend time in the area lacking stimulation [effect of compartment *F*_(2,8)_ = 70.9, *p* < 0.001 and day × compartment interaction *F*_(4,16)_ = 6.90 *p* = 0.002, **p < 0.01, ***p < 0.001 neutral vs paired compartments (Extended Data Fig. 8-1*Iii*); effect of compartment *F*_(2,2)_ = 54.2, *p* = 0.018, *p < 0.05 neutral vs paired compartments (Extended Data Fig. 8-1*Iiii*)]. In the current setups, optogenetic VTA-stimulation of DAT-Cre mice thus leads to place preference while same stimulation of Vglut2-Cre mice causes an avoidance to any compartment that activates photostimulation within the VTA.

10.1523/ENEURO.0066-19.2019.f8-1 Extended Data Figure 8-1 Complementary data from behavioral optogenetics. DAT-Cre-negative mice injected with *DIO-ChR2-eYFP* (***A***) and DAT-Cre-positive mice injected with *DIO-eYFP* mice (***B***) did not show preference to the light paired compartment so their results were pooled together (***C***). ***D***, Schematic illustration of optical fiber placement for mice analyzed in RT-PP analysis. ***E–G***, RT-PP testing under high power (20 mW, 5 ms, 20 Hz) stimulation for DAT-Cre/ChR2 (***E***), Calb2-Cre/ChR2 (***F***) and NEX-Cre/ChR2 (***G***). ***H***, RT-PP results for bilaterally injected NEX-Cre/ChR2 mice. ***A–C***, ***E–H***, Graphs: *p < 0.05, **p < 0.01, ***p < 0.001 paired versus unpaired; bar graphs: *p < 0.05, ***p < 0.001 versus paired, #*p* < 0.05, ###*p* < 0.001 versus unpaired. ***I***, Vglut2-Cre/ChR2 tested in the NCP test. ***Ii***, Schematic of the experimental setup. ***Iii–Iiii***, Mice spent significantly more time in the light-unpaired neutral compartment (*p < 0.05, **p < 0.01, ***p < 0.001 neutral vs Paired1 and Paired2 compartments). DAT-Cre negative *N* = 3, DAT-Cre/eYFP = 3, DAT-Cre/ChR2 high power *N* = 4, Calb2-Cre/ChR2 high power *N* = 7, NEX-Cre/ChR2 *N* = 4, NEX-Cre/ChR2 bilateral *N* = 4. NS, non-significant. Download Figure 8-1, EPS file.

#### Analysis of RT-PP and CR in Calb2-Cre and NEX-Cre mice

Using these behaviors as references and for comparison in the place preference setup, Calb2-Cre mice showed a strikingly different behavior. Neither preference nor avoidance was detected but instead, mice spent equal amount of time in both main compartments [effect of compartment *F*_(2,12)_ = 27, *p* < 0.001, day × compartment interaction, *F*_(12,72)_ = 1.45, *p* = 0.163 and no differences between paired versus unpaired across days (Fig. 8*D*, left); effect of compartment *F*_(2,6)_ = 90.1, *p* < 0.001, no differences between paired vs unpaired, ***p < 0.001, ###*p* < 0.001 neutral vs paired and unpaired, respectively (Fig. 8*D*, right)]. When analyzing whether optogenetic activation of NEX-Cre VTA neurons would cause place preference, a significant behavioral response toward the photostimulation was observed (effect of compartment *F*_(2,8)_ = 76.8, *p* < 0.001, day × compartment interaction, *F*_(12,48)_ = 4.63, *p* < 0.001; Fig. 8*E*, left). NEX-Cre mice responded weakly to VTA-photostimulation on days 3 and 4, but on days 6 and 7, NEX-Cre mice preferred the light-paired compartment (*p = 0.02, ***p < 0.001 paired vs unpaired). However, no CR was observed on either day 5 or 8 (Fig. 8*E*, left). By averaging the results of all four RT-PP days, NEX-Cre mice showed a significant preference for paired over unpaired and neutral compartments (effect of compartment *F*_(2,6)_ = 39.7, *p* < 0.001 *p = 0.013 ***p < 0.001 vs paired, ##*p* = 0.008 neutral vs unpaired; Fig. 8*E*, right).

#### Analysis of RT-PP and CR in DAT-Cre, Calb2-Cre, and NEX-Cre mice using higher power stimulation

While the result above demonstrated that activation of NEX-Cre VTA neurons induced place preference behavior, higher power stimulation (5-ms pulse width, 20 Hz, 20 mW) was subsequently used to test whether these laser parameters would boost the observed behavioral response. Again, DAT-Cre mice showed a strong preference for the light-paired chamber [effect of compartment *F*_(2,6)_ = 105, *p* < 0.001, day × compartment interaction *F*_(12,36)_ = 22.6, *p* < 0.001, ***p < 0.001 paired vs unpaired (Extended Data Fig. 8-1*E*, left); effect of compartment *F*_(2,6)_ = 404, *p* < 0.001, ***p < 0.001 unpaired and neutral vs paired (Extended Data Fig. 8-1*E*, right)], while Calb2-Cre mice continued not to respond to the VTA photostimulation [effect of compartment *F*_(2,12)_ = 12.5, *p* = 0.001, day × compartment interaction *F*_(12,72)_ = 0.469, *p* = 0.927 (Extended Data Fig. 8-1*F*, left); effect of compartment *F*_(2,6)_ = 47.3, *p* < 0.001, ***p < 0.001 and ###*p* < 0.001 neutral versus unpaired and paired (Extended Data Fig. 8-1*F*, right)]. In contrast, NEX-Cre mice showed a significant preference also with this higher power stimulation [effect of compartment *F*_(2,6)_ = 48.3, *p* < 0.001, day × compartment interaction *F*_(12,36)_ = 8.58, *p* < 0.001, **p = 0.003, ***p < 0.001 paired vs unpaired (Extended Data Fig. 8-1*G*, left); effect of compartment *F*_(2,6)_ = 178, *p* < 0.001, ***p < 0.001 paired vs unpaired and neutral, ###*p* < 0.001 unpaired vs neutral (Extended Data Fig. 8-1*G*, right)]. Finally, to further validate the role of NEX-Cre VTA neurons in place preference, a subset of NEX-Cre mice was bilaterally injected with *DIO-ChR2-eYFP* and tested in the same protocol under normal and high-power light stimulation (Extended Data Fig. 8-1*H*; Fig. 8*F*). Mice preferred the light-paired side over the unpaired under both conditions, and high-power stimulation accentuated the preference toward the light paired compartment which reached a 3-fold increase compared to the unpaired [standard power: effect of compartment *F*_(2,6)_ = 43.3, *p* < 0.001, day × compartment interaction *F*_(12,36)_ = 2.13, *p* = 0.04 (Extended Data Fig. 8-1*H*, left); effect of compartment *F*_(2,6)_ = 331, *p* < 0.001, ***p < 0.001 vs paired ###*p* < 0.001 vs unpaired (Extended Data Fig. 8-1*H*, right); high power: effect of compartment *F*_(2,6)_ = 36.5, *p* < 0.001, day × compartment interaction *F*_(12,36)_ = 9.03, *p* < 0.001, ***p < 0.001 paired vs unpaired (Fig. 8*F*, left); effect of compartment *F*_(2,6)_ = 106 *p* < 0.001, ***p < 0.001 paired vs unpaired and neutral, #*p* = 0.011 unpaired vs neutral (Fig. 8*F*, right)]. However, unlike DAT-Cre mice, NEX-Cre mice did not show any CR in any RT-PP experiment [day 5 paired vs unpaired *p* > 0.999, day 8 paired vs unpaired *p* = 0.937 (Fig. 8*E*); day 5 paired vs unpaired *p* > 0.999, day 8 paired vs unpaired *p* = 0.989 (Fig. 8*F*)].

## Discussion

It is well established that the VTA is involved in a range of functions, including behavioral reinforcement, reward, aversion, motivation and incentive salience (Morales and Margolis, 2017). However, an area of active investigation is how the VTA can possess the ability to contribute to all of these diverse functions, some even contrasting. It is now becoming increasingly clear that functional diversity within the mDA system might be matched by molecular and anatomic heterogeneity (Lammel et al., 2011, 2012; Roeper, 2013; Morales and Margolis, 2017; Poulin et al., 2018). Why is this important? The possibility to determine the exact identity of neurons that contribute to a particular behavior opens up entirely new perspectives in the opportunity to to selectively target only those neurons that contribute to clinical symptoms without causing side-effects by affecting adjacent neuronal populations. In this study, we used Cre-driven mouse genetics and optogenetics to begin to disentangle the contribution of the newly described NeuroD6 VTA subtype (Viereckel et al., 2016; Khan et al., 2017; Kramer et al., 2018) in reward-related behaviors commonly ascribed to the VTA DA system. The main finding of our study is that despite their restricted number, NeuroD6 VTA neurons contribute to psychostimulant-induced hyperlocomotion and that their activation induces place preference behavior.

### NeuroD6 VTA neurons represent a modest neuronal population within the VTA with molecular capacity for dopaminergic and glutamatergic neurotransmission

In the current study, we showed that NeuroD6 VTA neurons constitute a modest proportion (circa 12%) of all VTA neurons expressing the gene encoding TH within the PN, PIF, PBP, IF, and RLi subareas. Within these VTA subareas, all NeuroD6-positive neurons were positive for both Th and Dat mRNAs, markers of dopaminergic neurons. In addition, while no or very few NeuroD6 neurons were positive for Viaat mRNA, a marker of GABAergic neurons, 12% of the NeuroD6/Th double-positive neurons within the VTA were positive for Vglut2 mRNA, suggesting a capacity for dual dopaminergic/glutamatergic neurotransmission. Indeed, DA/glutamate co-release has in several studies been shown as a property of certain mDA neurons where it has been proposed to play a role in reward-related behavior reinforced by DA (for recent review, see Trudeau and El Mestikawy, 2018). The identification of co-labeling of NeuroD6 mRNA with Th, Dat and Vglut2 mRNAs within distinct neurons was partly in accordance with our analysis of a NEX-Cre transgenic mouse line, implemented here to achieve manipulation of the NeuroD6 VTA neurons, which identified substantial co-localization between NEX-Cre-driven reporter gene expression (YFP) and TH immunofluorescence. However, lack of TH/YFP co-localization was also identified. The findings showing that the majority of NeuroD6 VTA neurons expressed DA markers were in accordance with our electrophysiological data in which optogenetic VTA stimulation of NEX-Cre neurons enabled the identification of DA release, as further discussed below. Further, optogenetic stimulation also gave rise to EPSCs of glutamatergic nature, while no GABAergic currents were detected, in agreement with the co-localization of NeuroD6 mRNA with Vglut2 mRNA but lack of significant co-localization with Viaat mRNA.

In the context of transgenic mice, it is noteworthy that our result showing non-complete overlap between NEX-Cre-driven reporter gene expression and TH, which contrasts the parallel finding that all VTA neurons positive for endogenous NeuroD6 mRNA also label for Th mRNA, are in accordance with a recent study in which a substantial number of non-dopaminergic NEX-Cre VTA neurons were identified (Kramer et al., 2018). Collectively, these findings propose that interpretation of VTA-data originating from the current NEX-Cre mouse line should be considered with awareness of complex downstream neurocircuitry. Further, as extensively discussed in the literature, regulatory promoters implemented experimentally to drive Cre expression may give rise to transient and/or ectopic Cre activity that fails to mimic endogenous gene expression due to gene regulatory events, not least during developmental phases. Indeed, patterns of ectopic Cre activity have been described for other transgenic mouse lines, including DAT-Cre and TH-Cre transgenic mouse lines commonly implemented for the study of DA neurons (Lindeberg et al., 2004; Stamatakis et al., 2013; Lammel et al., 2015; Nordenankar et al., 2015; Pupe and Wallén-Mackenzie, 2015; Stuber et al., 2015; Morales and Margolis, 2017). While the current NEX-Cre transgenic line has been thoroughly validated recently for the study of VTA neurons (Khan et al., 2017; Kramer et al., 2018), to direct selectivity to VTA DA neurons, we here implemented a conditional genetic approach to specifically abrogate vesicular packaging of DA in NEX-Cre neurons. Further, we used optogenetically driven neuronal activation to study effects upon direct stimulation of NEX-Cre VTA neurons.

### Targeting of the *Vmat2* gene in NEX-Cre VTA DA neurons allowed identification of a role in psychostimulant-mediated response

To enable the study of how reward-related behaviors classically associated with the mDA system would be affected if the NEX-Cre DA neuron subtype lost its ability for dopaminergic function, a conditional gene-targeting approach was implemented in which VMAT2 was ablated specifically from NEX-Cre neurons. Since we could show that NeuroD6 and Vmat2 mRNAs only co-localized within the VTA, no other monoaminergic population should suffer from loss of VMAT2 by this approach. Indeed, the results confirmed that Vmat2 mRNA was selectively knocked out within the VTA, while all other monoaminergic neurons maintained normal Vmat2 mRNA. Thus, the *Vmat2^lox/lox;NEX-Cre^* mouse line forms a new mouse model of DA-release deficiency from a restricted group of VTA DA neurons characterized by NeuroD6 promoter activity. Based on the importance of mDA system in processing natural and drug rewards (Kalivas et al., 1992; Di Chiara and Bassareo, 2007; Ikemoto, 2007; Baik, 2013; Robinson and Berridge, 1993), we addressed the behavioral responses of *Vmat2^lox/lox;NEX-Cre-tg^* cKO mice and *Vmat2^lox/lox;NEX-Cre-wt^* control mice to sugar, ethanol and the psychostimulants amphetamine and cocaine. cKO mice displayed higher locomotor activation on repeated administration of psychostimulants than control mice. In contrast, sugar preference and CPP to cocaine and amphetamine were similar between cKO and control mice, and both genotype groups showed a preference for increasing dose of ethanol, albeit in different patterns.

While acute administration of cocaine failed to cause differences in locomotor responses between cKO and control mice, repeated administration caused exaggerated locomotor behavior in cKO mice when measured in the CPP paradigm. In contrast, with repeated amphetamine injections, the locomotor response was elevated above control levels in the open field, but not in the CPP. The tests implemented were designed to study different behavioral parameters, and results obtained in different setups and by different drugs are therefore not directly comparable. What may seem as apparent discrepancies might be related to several different properties. Firstly, the size and properties of the test environment were substantially different between setups. The open field test took place in an environment that resembled the home cage. Locomotion was recorded during the conditioning phase when the mice were confined to a much smaller compartment with specific patterns and no bedding. Secondly, the injection regime differed between tests. In the open field, mice received acute injections of cocaine or were sensitized to amphetamine by receiving daily injections after a 30-min habituation period. In contrast, in the CPP experiment, the mice received in total four injections of the drug in two non-consecutive days without any previous habituation period. Finally, the recording period was shorter in the CPP compared to the open field (30 min vs 1.5 h), a parameter that could mask the long-lasting effects of amphetamine on locomotion. Further, the observation of heightened, rather than reduced, psychostimulant-induced locomotion might seem counter-intuitive: Loss of VMAT2 should lead to decreased packaging and release of DA which might be expected to cause reduced locomotion compared to control levels. However, the results obtained from our spatially selective cKO mice are similar to the heightened amphetamine-induced hyperlocomotion observed in a study of mice heterozygous for *Vmat2* in all DAT-Cre neurons (Isingrini et al., 2016). Thus, lowering the level of VMAT2 throughout all DAT-Cre neurons or ablating it within the NEX-Cre VTA DA population give rise to similar behavioral consequences. Further analyses focused around VMAT2 in psychomotor behavior will be necessary to pin-point this matter, however, developmental adaptations, a common feature of KO strategies induced during embryonal development, may underlie the heightened locomotor response.

### Striatal optogenetic stimulation in NEX-Cre mice induced DA release and glutamatergic EPSCs

Complementary to the cKO approach, we used optogenetics-based experiments in which the NEX-Cre VTA population could be directly stimulated. This type of manipulation provides high spatial and temporal resolution (Deisseroth, 2015) and thus has the advantage of enabling selective stimulation of Cre-driven neurons in real time with the benefit of directly pin-pointing the role of molecularly defined neurons in measurable behavior. By analysis of optogenetic reporter gene (eYFP) expression upon injection into the VTA of NEX-Cre mice, we showed that NeuroD6 VTA neurons projected mainly to the NAcSh of the striatal complex, with substantially lower density than observed upon similar injection in DAT-Cre and Vglut2-Cre mice used here as controls (Stuber et al., 2010; Hnasko et al., 2012; Pascoli et al., 2015; Qi et al., 2016; Yoo et al., 2016). NEX-Cre VTA projections also reached several additional areas, but with even lower density than seen in the NAcSh, including the OT, medial habenula and ventral pallidum. In accordance with the co-localization of eYFP with TH immunoreactivity, we could verify that NEX-Cre VTA neurons released DA in both the NAcSh and OT upon striatal optogenetic stimulation. Although the levels were lower than those observed upon similar stimulation of DAT-Cre-positive VTA neurons, they were significantly higher than those observed in control experiments, demonstrating that the NEX-Cre VTA population indeed releases measurable amounts of DA in their target areas. To investigate whether the TH-negative cellular population, present most profoundly in the medial VTA, was of glutamatergic or GABAergic nature, patch-clamp electrophysiology was performed which showed that optogenetic stimulation of NEX-Cre terminals induced EPSCs, but not IPSCs, in NAcSh, thus verifying glutamatergic neurotransmission. While glutamatergic postsynaptic currents were evidently a result of the optogenetic stimulation of NEX-Cre VTA neurons, it remains to be established if the rare endogenous NeuroD6+/Th+/NeuroD6+ triple-positive neurons observed in our histologic analysis are sufficiently potent to drive a similar postsynaptic response in the natural situation, that is, upon excitation of the NeuroD6 VTA neurons in a non-transgenic context. Finally, the current setup did not allow us to conclude if the EPSCs were of monosynaptic or polysynaptic nature. The short onset of EPSCs was suggestive of monosynaptic transmission, however, electrophysiological approaches combined with pharmacological agents will be necessary to fully define the signaling properties.

### Optogenetic stimulation of NEX-Cre VTA neurons reveals a role in place preference behavior

Optogenetic stimulation of the mDA system of TH-Cre and DAT-Cre mice has been demonstrated to potently induce DA release and real time place preference (Tsai et al., 2009; Stuber et al., 2010; Yoo et al., 2016). The same type of activation of VTA in Vglut2-Cre mice has been described to cause postsynaptic glutamatergic currents and to induce either place preference or place avoidance, depending on stimulation parameters (Hnasko et al., 2012; Wang et al., 2015; Qi et al., 2016; Yoo et al., 2016). Using DAT-Cre and Vglut2-Cre mice as references, we could show here that optogenetic stimulation within the VTA of NEX-Cre mice induced a significant preference for the light-paired compartment. The magnitude of the preference observed was, however, smaller in NEX-Cre than in DAT-Cre mice. This difference is likely related to the substantially smaller population of VTA neurons activated upon photostimulation in the NEX-Cre compared to DAT-Cre VTA and the different projection patterns of these neuronal populations. This is supported by the analysis of YFP-positive fibers, which differ substantially between DAT-Cre and NEX-Cre mice. VTA-injection of ChR2-YFP in DAT-Cre mice results in strong YFP-fluorescence in all innervation areas ascribed to the mDA system. In contrast, the same injection into the VTA of NEX-Cre mice results in substantially lower YFP-derived fluorescence in the VTA and sparse fluorescence in target areas.

Despite smaller magnitude, the ability of NEX-Cre VTA neurons to induce real time place preference is an important finding as it demonstrates the possibility of identifying spatially restricted groups of VTA neurons that are sufficient to induce a measurable behavior. Further arguing for the importance of this result, the optogenetically induced preference behavior displayed by NEX-Cre mice was strengthened by viral injections in bilateral, rather than unilateral, manner as well as by increased laser power. The results of these experimental manipulations suggest that the enhanced recruitment of NEX-Cre neurons strengthened the behavioral output. While additional studies will be required to completely disentangle the behavioral role of NeuroD6 VTA neurons, the current optogenetics-based setup already enabled us to demonstrate that VTA activation in NEX-Cre mice could induce place preference in real-time, but that it failed to result in CR, defined as significant place preference even in absence of actual optogenetic stimulation. This contrasts the strong CR observed in the DAT-Cre mice, and hence, activation of VTA populations in NEX-Cre mice and DAT-Cre mice differ in more than one parameter: Magnitude in real time place preference and presence of a detectable CR. In contrast to the preference behavior displayed by NEX-Cre and DAT-Cre mice, optogenetic stimulation of VTA Vglut2-Cre neurons led to real time place avoidance defined here as reduced time spent in the stimulation-paired compartment. This result is consistent with a recent study which found that real time avoidance coincided with a frequency-dependent increase in entries to the light-paired compartment and robust self-stimulation in an operant task (Yoo et al., 2016). In contrast, another study found that photostimulation of Vglut2-Cre neurons in VTA induced modest real time place preference and self-stimulation (Wang et al., 2015). These data show that the behavioral effects of VTA glutamate neuron stimulation are sensitive to the task, including the design of the apparatus and stimulus parameters. In this context, it is noteworthy that VTA neurons of the NEX-Cre transgenic mouse line, with their mixture of dopaminergic and glutamatergic signaling properties, might have shown lower level of place preference than DAT-Cre mice not only due to the smaller number of neurons and sparser projections, but also as their activation might have caused a glutamate-mediated avoidance behavior that counterbalanced the behavioral preference for light stimulation.

### NeuroD6 and Calb2 mRNAs show partial overlap, but NEX-Cre and Calb2-Cre VTA neurons have distinct projections and role in behavior

Parallel to the focus on NeuroD6 VTA neurons in neurocircuitry and behavioral regulation, our histological analysis enabled us to identify a degree of co-localization between NeuroD6 and Calb2 mRNAs. While NeuroD6 mRNA was uniquely found in the VTA and excluded from the SNc, Calb2 mRNA was found distributed throughout these dopaminergic areas. However, histological analysis showed a degree of co-localization between NeuroD6 and Calb2 mRNAs, a finding which adds to the recent molecular description of NeuroD6 as co-localized with gastrin-releasing peptide (GRP) and additional markers (Khan et al., 2017; Kramer et al., 2018; Poulin et al., 2018). Beyond the partial co-localization of NeuroD6 and Calb2 mRNAs, the results demonstrate that Calb2 VTA neurons constitute a substantially larger proportion within the mDA population, show considerable expression of the gene encoding VIAAT, and are present in the SNc, an area devoid of NeuroD6 neurons. Our neurophysiological circuitry analyses of Calb2-Cre mice showed that Calb2-Cre VTA neurons belong to the category of VTA/SNc neurons that projects to the OT where their stimulation resulted in DA release and glutamatergic postsynaptic currents. While it was recently described that activation of dopaminergic fibers from VTA to the medial OT can induce place preference in DAT-Cre mice (Zhang et al., 2017), a similar response was not observed here on Calb2-Cre VTA stimulation. These differences might be explained by the difference in density of the innervation patterns in the OT between the DAT-Cre and Calb2-Cre mice. The difference in preference behavior between NeuroD6-Cre and Calb2-Cre mice shows that distinct VTA neurocircuitry is crucial for the behavioral output.

### Unraveling the behavioral roles of NeuroD6 VTA neurons stands to benefit current decoding of VTA-related disorders

The behavioral complexity mediated by the VTA is implicated in a range of neuropsychiatric conditions including substance use disorder, schizophrenia, and ADHD for which clinical interventions based on increasing, decreasing, stabilizing or modulating the mDA system are commonly prescribed. In addition, since VTA DA neurons are less susceptible to degeneration in PD than SNc DA neurons, molecular differences are intensively searched for. GRP, in several studies identified as a marker for VTA DA neurons (Chung et al., 2005; Greene et al., 2005; La Manno et al., 2016; Viereckel et al., 2016) was recently shown to co-localize with NeuroD6 (Kramer et al., 2018). Several lines of evidence suggest that a discrete NeuroD6/GRP VTA subtype should be of specific interest. Overexpression of the gene encoding GRP increased the survival rate of cultured DA neurons in a parkinsonian experimental model (Chung et al., 2005) and GRP-positive mDA neurons remain in biopsies from deceased PD patients (Viereckel et al., 2016). Further, NeuroD6 increases neuronal survival in a toxin model of PD (Kramer et al., 2018). The NeuroD6/GRP VTA subtype might thereby possess resistance to PD. Our current results show that, despite their modest representation within the VTA, NeuroD6-expressing VTA neurons are implicated in distinct aspects of reward-related behavior. Their resistance to PD may thus contribute to the cause of behavioral dysfunction observed in the non-motor symptom domain of PD, including treatment-induced complications that resemble aspects of neuropsychiatric diseases, such as behavioral addictions (Cenci et al., 2015).

Current molecular profiling of DA neuron subtypes should prove valuable for prospects of selective treatment in conditions related to VTA dysfunction. Of essence to achieve such selectivity is the systematic decoding of the explicit behavioral roles mediated by distinct VTA neurons. In this study, we initiated such analysis and now propose that NeuroD6 VTA neurons are of particular interest for further analysis of motivated and addictive behavior as they are here implicated in reward-related behavior measured as real time place preference and as their controlled dysregulation alters the responsiveness to psychostimulants. Our findings should prove useful for future investigations aimed at advancing the knowledge of VTA neurocircuitry in healthy conditions and in neuropsychiatric illness implicating the VTA.

## Synthesis

Reviewing Editor: Yavin Shaham, NIDA-IRP/NIH Decisions are customarily a result of the Reviewing Editor and the peer reviewers coming together and discussing their recommendations until a consensus is reached. When revisions are invited, a fact-based synthesis statement explaining their decision and outlining what is needed to prepare a revision will be listed below. The following reviewer(s) agreed to reveal their identity: David Root.

The reviewers found merit in the study but also have raised several significant concerns that should be addressed in the revision. It is particularly important to satisfactory address comments 1-2 of review 2. Note that while the reviewers made suggestions for additional behavioral experiments, these are not mandatory for a successful revision. I enclose below the individual comments of the reviewers and in addition to their comments, also address the comments below:

1. Based on the reviewers' comments, it is important to tone down the different conclusions and statements in the text regarding the role of NeuroD6 in reward.

2. Lines 7 and 29: change “not least addiction” to “including drug addiction”

3. Line 20-24: please describe in concrete terms what “altered locomotor response” means. Did the experimental manipulation decrease or increase the locomotor response to the drugs? Also, change “is sufficient to drive significant” to ‘caused’ and ‘fails’ to ‘failed’ (in general the findings described in the paper should be described in past tense).

4. Abstract: please rewrite this section to also include the negative results in the study

5. Methods: please provide RRID numbers of the different antibodies and other reagents when possible. Also, change ‘animals’ to ‘mice’ when referring to the experimental animals

6. Results: please add F or t values and their degrees of freedom to all p values reported in the Results section. Also, please describe the different behavioral results under different sub-headings in different paragraphs. Finally, please remove background text with literature citations and Methods-related text from this section. The background text belongs to either the Introduction or Discussion and the Methods text belongs to the Methods section.

7. Results, DA release section and subsequent text below it: please add F values, degrees of freedom, and p values for descriptions of group differences

8. Line 59 and other places in the text: Change “is sufficient to drive place preference” to ‘induced’ or ‘caused’

9. Discussion: please add 2-3 informative sub-headings to orient the readers of the topics being discussed. In line 741, change “strikingly heterogeneous” to “heterogeneous”; in general, avoid using subjective terms to describe experimental findings, these are not necessary

10. Figure legends 6-7: add the n of mice per group

11. Figure 1c-d: change the y-axis to start from 0%

REVIEWER 1

NeuroD6-expressing DA neurons were recently identified as neuroprotected in experimental Parkinsons. However, the circuits and functions of NeuroD6 neurons are not entirely known. The authors used multiple mouse lines to identify the circuits and functions of NeuroD6 VTA neurons. Many experiments are conducted, most with null effects. However, it is identified that NeuroD6 neurons release dopamine into accumbens and can drive conditioned reward when optogenetically stimulated. The experiments identify important insights into VTA heterogeneity and function. However, as written, there are a few inconsistencies in the data that require further explanation prior to publication. These major concerns are listed below:

Major concerns:

1. The removal of the capacity to vesicularly release dopamine from NeuroD6 neurons enhanced sensitization to amphetamine but not to cocaine. In addition, the enhanced locomotion to amphetamine was not replicated at the same dose with the CPP procedure. This raises the question of how reliable the original amphetamine finding is.

2. The authors write that a few NeuroD6 cells co-expressed VGluT2 but in Fig 1 it is stated that 100% of NeuroD6 cells lack VGluT2. In addition, how would it be possible for VGluT2-lacking neurons to cause glutamatergic currents (Calb and NEX)?

3. Calb and NEX release glutamate in OT and shell. Are the currents monosynaptic? Testing under TTX and 4-AP could test this and determine if glutamate release is polysynaptic.

4. It was unclear if NEX::Cre-labeled neurons express NeuroD6. 74% of labeled neurons have TH protein, but all NeuroD6 neurons have TH mRNA. This dichotomy should be explained.

5. It is a little puzzling why Calb2::cre mice did not show dopamine release when most of these cells are TH+. Do they lack VMAT2?

6. Some NEX-eYFP labeling was observed in interpeduncular nucleus. Was this a projection or cell body labeling outside VTA? This would be consistent with medial habenula labeling (Table 2)

7. Stimulation of VTA VGluT2 cell bodies caused aversion, which differs from previous reports of reward (though aversion at terminal regions). The authors write that there were differences in procedure but were not fully explained that might mediate the observed difference.

Minor Concerns:

1 (Line 59)...omit “over the past couple of years”

2 (Line 10)...omit ‘e.g’

3 Missing figure label for Figure 2 (No description for E)

4 Line 401: that, not than

5 It should be noted that the ChR2-eYFP outputs identified in the paper are plausible outputs as axons are shown but not necessarily synapses.

REVIEWER 2

The authors use a combination of in situ hybridization, as well as transgenic and viral approaches to characterize and manipulate selective populations of midbrain dopamine neurons with a particular focus on NeuroD6-expressing neurons. They then employ voltammetry, slice electrophysiology, and behavioral analysis to interrogate the functional properties of this neuron subpopulation. The manuscript is well written and the purpose of the study - to further tease apart functional roles of molecularly distinct groups of dopamine neurons - has great merit. However, I have concerns about several of the technical approaches used and don't feel that the data currently support the claims that the manuscript advances regarding the role of NeuroD6+ dopamine neurons in reward-related behaviors. The conclusions presented need to be much more conservative in their wording.

Major concerns:

1) Although several published studies have characterized the abundance and spatial distribution of NeuroD6 neurons, additional confirmation would still be useful and it's good that the authors carry out their own analysis of the NeuroD6+ neuron abundance and distribution. However, the in situ images they present are not convincing. Figure 1A is labeled and described in the figure legend as showing TH mRNA in green. But there many red cells visible and some that appear violet as though there is also colocalization with a third marker or DAPI. Then in panel 1B, which is supposed to show NeuroD6 (red) and TH (green), the same green TH signal from panel 1A is shown, but a different population of red cells is visible. The red signals that are visible are also readily apparent in the SNc and many of them are TH-negative. This does not match with the statement that cells expressing NeuroD6 mRNA are only located in the medial VTA and that 100% of those cells are TH+. Panel 1C is supposed to show NeuroD6 only, but this is a different set of red signals than those that were shown in panel 1B and the signal is very dim. The authors should double check to see if there has been some mix up in which images were included in figures. The authors should also show higher magnification images of the medial VTA that clearly illustrate 100% overlap of NeuroD6 mRNA with TH mRNA in many cells rather than one tiny inset showing one cell. I have similar concerns about panel 1E where there are appear to be abundant red cells not colocalized with DAT mRNA, yet the claim is that 100% of NeuroD6 cells are DAT+. In panel 1G, it looks like there are many yellow cells positive for both NeuroD6 and Viaat, but the claim is that all NeuroD6 cells are Viaat negative. Higher magnification images of the medial VTA rather than tiny insets could help to clarify all of these concerns. Because these data are the foundation for most other analyses and interpretation that follows in the manuscript, it is critical for this piece of the dataset to be absolutely convincing.

2) The approaches used to confirm Vmat2 knockout in NeuroD6 neurons seem unnecessarily indirect and thus raise questions. Why not just quantify overlap between NeuroD6 mRNA and probe 2 for Vmat2 mRNA? With the current data, all we know is that Vmat2 is eliminated from some neurons in the medial VTA. We don't have any information about whether this Vmat2 elimination was specific to NeuroD6 neurons (and it likely isn't based on their findings with ChR2-EYFP expression in Nex-Cre mice in Figure 5). We also don't know from what percentage of NeuroD6 neurons was Vmat2 eliminated. If for some reason the authors can't look directly at overlap between NeuroD6 mRNA and probe 2 for Vmat2, they could at least quantify the number of Vmat2 probe2+probe1- cells in the different sub regions of the VTA and see how closely this matches the number and density of NeuroD6+ cells in those same sub regions. Again, this is part of the foundation for claiming that NeuroD6 neurons play a unique role in reward-related behaviors, so these data should be absolutely convincing for clear interpretation of behavioral experiments that follow.

3) There seems to be enough non-specific expression of ChR2 in the Nex-Cre mice that this complicates interpretation of the behavioral experiments. There were numerous EYFP+TH- cells in Nex-Cre mice. Further, although there was virtually no overlap between NeuroD6 mRNA and VGlut2 mRNA in figure 1 in situ experiments, clear glutamatergic currents were recorded in the NAc shell upon stimulation of ChR2+ terminals in these mice. VTA optogenetic stimulation in these mice during behavior experiments is very likely resulting in activation of NeuroD6 dopamine neurons and some VTA glutamatergic neurons. For this reason, the authors should be more cautious in their claims about potential roles for NeuroD6 dopamine neurons in reward-related behaviors. They can say that their experiments suggest roles for these neurons in reward-related behaviors but need to be very clear that additional experiments are needed to unequivocally establish which behavioral effects are due to NeuroD6 neurons alone. Perhaps using a viral construct with DAT promoter (or some other dopamine neuron-specific promoter) driving DIO-ChR2-EYFP could be a viable approach?

Minor concerns:

1) A number of the behavioral results are inconsistent or counter-intuitive. For example, there isn't a consistent impact of Nex-Cre Vmat2 knockout on psychostimulant induced locomotor behavior (different effects for cocaine versus amphetamine and different effects during open field analysis compared to during CPP). The authors delve into this a bit in the discussion, but this is another reason why the claims advanced in the abstract and title need to be softened. It seems a bit surprising to me that you could see a clear effect of ChR2 stimulation in Nex-Cre mice on real time place preference but no impact on conditioned place preference and no impact of Vmat2 deletion on conditioned place preference. I would have liked to see more discussion of how the behavioral analysis in Vmat2 knockout and ChR2 stimulation in Nex-Cre mice relate to one another. Behavioral analysis is not my main area of expertise so hopefully other reviewers can add additional comments to this portion of the study.

2) Many of the higher magnification insets throughout the manuscript are quite pixelated. Increased image resolution will make the images easier to interpret.

3) FIGURE 4 - why are 83% of Calb2 neurons positive for TH and only 51% are positive for DAT. This seems a little strange.

4) Fig4E - where is the location of the inset?

5) Text line 522 states that the percent overlap between YFP and TH exceeds that of the quantification from mRNA and so they moved forward with analysis of neurotransmitter release; but my understanding was that fewer YFP+ cells were positive for TH than NeuroD6 cells were positive for TH (NEX-Cre: 74% YFP+ are TH+ versus 100%NeuroD6 are TH+ at messenger RNA level; Calb2-Cre: 67% YFP+ are TH+ versus 81%Calb2 are TH+ at messenger RNA level).

6) Fig7A - ideally show the recording site for OT as well otherwise this is confusing and makes you think that recording site is NAc shell for both Cre lines.

7) Fig7C - is a bit confusing - the y axis is showing amplitude of EPSCs but then x axis says % connectivity and two numbers are shown. I am assuming these represent a range of % of cells that showed currents? It might be more clear to make a separate graph for percent connectivity or just state the percent connectivity in the results.
